# TCF3 is epigenetically silenced by EZH2 and DNMT3B and functions as a tumor suppressor in endometrial cancer

**DOI:** 10.1038/s41418-021-00824-w

**Published:** 2021-06-26

**Authors:** Tao Gui, Ming Liu, Bing Yao, Haiqin Jiang, Dongjun Yang, Qixiang Li, Xiangwei Zeng, Ying Wang, Jian Cao, Yexuan Deng, Xinyu Li, Peipei Xu, Liqin Zhou, Dake Li, Zhihui Wang, Ke Zen, David C. S. Huang, Bing Chen, Guiping Wan, Quan Zhao

**Affiliations:** 1 The State Key Laboratory of Pharmaceutical Biotechnology, Department of Hematology, the Affiliated Drum Tower Hospital of Nanjing University Medical School, China-Australia Institute of Translational Medicine, School of Life Sciences, Nanjing University, Nanjing, China; 2 Department of Obstetrics and Gynecology, Affiliated Hospital of Integrated Traditional Chinese and Western Medicine, Nanjing University of Chinese Medicine, Nanjing, China; 3 Women’s Hospital of Nanjing Medical University (Nanjing Maternity and Child Health Care Hospital), Nanjing, China; 4 Department of Medical Genetics, Nanjing Medical University, Nanjing, China; 5 Department of Obstetrics and Gynecology, Suzhou Xiangcheng People’s Hospital, Suzhou, China; 6 The Walter and Eliza Hall Institute of Medical Research, Department of Medical Biology, University of Melbourne, Melbourne, VIC Australia

**Keywords:** Tumour-suppressor proteins, Epigenetics

## Abstract

Endometrial cancer (EC) is the most common gynecological malignancy worldwide. However, the molecular mechanisms underlying EC progression are still largely unknown, and chemotherapeutic options for EC patients are currently very limited. In this study, we found that histone methyltransferase EZH2 and DNA methyltransferase DNMT3B were upregulated in EC samples from patients, and promoted EC cell proliferation as evidenced by assays of cell viability, cell cycle, colony formation. Mechanistically, we found that EZH2 promoted EC cell proliferation by epigenetically repressing TCF3, a direct transcriptional activator of CCKN1A (p21^WAF1/Cip1^), in vitro and in vivo. In addition, we found that DNMT3B specifically methylated the TCF3 promoter, repressing TCF3 expression and accelerating EC cell proliferation independently of EZH2. Importantly, elevated expression of EZH2 or DNMT3B in EC patients inversely correlated with expression of TCF3 and p21, and was associated with shorter overall survival. We show that combined treatment with GSK126 and 5-Aza-2d treatment wit synergistically inhibited methyltransferase activity of EZH2 and DNMT3B, resulting in a profound block of EC cell proliferation as well as EC tumor progression in cell line-derived xenograft (CDX) and patient-derived xenograft (PDX) mouse models. These findings reveal that TCF3 functions as a tumor suppressor epigenetically silenced by EZH2 and DNMT3B in EC, and support the notion that targeting the EZH2/DNMT3B/TCF3/p21 axis may be a novel and effective therapeutic strategy for treatment of EC.

## Introduction

Endometrial cancer (EC) is the most common gynecological malignancy worldwide, with over 61,880 new cases and nearly 12,160 deaths estimated in 2019 in the United States [1]. Owing to their aggressive characteristics, serious ECs have high recurrence rates, and are associated with high mortality, having very poor five-year survival rates of less than 30% [1, 2]. Currently, standard treatment for all ECs is surgery followed by adjuvant therapy (chemotherapy and/or radiation therapy, etc.) based on grade and tumor stage [3]. However, to date, chemotherapeutic options for EC patients have been limited; FDA-approved targeted therapies for ECs include hormonal intervention (for hormone-dependent endometrioid ECs), the immune checkpoint inhibitor, pembrolizumab, and recently the combination of pembrolizumab with lenvatinib for the treatment of a special category of advanced ECs [4, 5]. In view of these limited options for targeted regimes for treating ECs, there is an urgency for revising and improving therapies for EC.

Accumulating evidence suggests that epigenetic alterations including aberrant DNA methylation and histone modifications play key roles in the occurrence and development of EC [6, 7]. To date, DNA promoter hypermethylation, which may contribute to carcinogenesis, has been identified in several target genes in endometrial cancer, including hMLH1, p53, PTEN [8]. On the other hand, trimethylation on lysine 27 of histone H3 (H3K27me3) triggered by enhancer of zeste homolog 2 (EZH2), a critical component of the polycomb repressive complex 2 (PRC2) that mediates gene silencing [9], has been shown to be associated with EC progression [10, 11]. However, the roles of EZH2 and DNMT3B and underlying molecular mechanisms for malignant transformation and cell proliferation in EC remain largely unknown, although increased EZH2 and DNMT3B expressions have been shown in EC tissues [11–14].

Here, we show that EZH2 and DNMT3B promote EC cell proliferation by epigenetically repressing TCF3, a transcriptional activator of CCKN1A (p21^WAF1/Cip1^), in vitro and in vivo. We demonstrate that combined treatment with GSK126 and 5-Aza-2’-deoxycytidine to inhibit methyltransferase activity of EZH2 and DNMT3B synergistically block EC cell proliferation and EC tumor progression in both cell line-derived xenograft (CDX) and patient-derived xenograft (PDX) mouse models. These findings may help provide a novel and effective therapeutic strategy for treating EC.

## Materials and methods

### Clinical samples, tissue array, and immunohistochemical (IHC) staining

Fresh matched EC tissues and adjacent normal tissues were derived from patients undergoing surgical procedures at the Jiangsu Provincial Hospital on Integration of Chinese and Western Medicine (Nanjing, China). All patients provided written informed consent for participation in this study. Study on fresh clinical samples was approved by the ethics committee of Jiangsu Provincial Hospital on Integration of Chinese and Western Medicine. The EC tissue microarrays (TMA, HUteA060CS01, containing 26 matched EC tissues and adjacent normal tissues collected from EC patients) were constructed by Shanghai Outdo Biotech Co., Ltd. (Shanghai, China). IHC staining was performed by Nanjing Microworld Biotechnology Co., Ltd. Slides were incubated with antibodies specific for EZH2 (CST, #5246, 1:300), TCF3 (CST, #12258, 1:200), p21 (CST, #2947, 1:100), DNMT3B (Abconal, A2889, 1:200), and Ki-67 (Abcam, ab15580, 1:400). Images were digitalized using an Aperio scanner. Immunohistochemical staining of interested proteins in the tissue was scored, according to the semi-quantitative H score, independently by two pathologists blinded to the clinical data [15]. Rare discordant scores were resolved by re-review of the slide and consultation between the pathologists. Staining of cells was analyzed according to staining intensity on a scale of 0–3 (0 = negative, weak = 1, moderate = 2, strong = 3). The H score was calculated by multiplying the scale score times the percent of cells having that score, and then summing the products across all scale scores, i.e., H score (0–300 scale) = 3 × (% at 3) + 2 × (% at 2) + 1 × (% at 1). The clinical features of the patients are listed in Supplementary Table S1. For survival analyses, patient overall survival was stratified by expression of the gene of interest, and is presented as Kaplan–Meier plots, and tested for significance using log-rank tests. Degrees of correlation between stained proteins for determining patient protein-expression patterns were assessed via Pearson correlation analysis.

### Cell lines and cell culture

Endometrial adenocarcinoma cell lines, ISK (well-differentiated), and KLE (poorly differentiated), used in the study were obtained from ATCC. The cells were cultured in RPMI1640 media (Life Technologies) containing 10% (v/v) FBS (Life Technologies) and 1% (v/v) penicillin/streptomycin. Cells were maintained in a 5% CO2 incubator at 37 °C and passaged every 2 or 3 days. Cell lines were routinely tested to exclude mycoplasma contamination and were recently authenticated by Genetic Testing Biotechnology Corporation (Suzhou, China) using short tandem repeat (STR) profiling.

### RNA isolation and qRT-PCR analysis

RNA from cell lines was isolated using Trizol reagent (Invitrogen). Synthesis of complementary DNAs (cDNAs) was done using HiScrip^®^ III RT SuperMix for qPCR (with gDNA wiper) (Vazyme Biotech). Quantitative reverse transcription PCR (qRT-PCR) was performed using AceQ qPCR SYBR Green Master Mix (Vazyme Biotech) according to the manufacturer’s protocols. Experiments were performed in a StepOnePlus^TM^ Real-Time PCR System (Applied Biosystems), and data were analyzed with StepOne Software v2.1 (Applied Biosystems). Relative mRNA levels of each gene were normalized to the expression of a reference gene GAPDH. The sequences of the primers for qRT-PCR are listed in Supplementary Table S3.

### Lentiviral production and transduction

HEK293T cells (5 × 10^6^ per plate) were seeded into 10 cm cell culture dishes and incubated overnight at 37 °C, 5% CO_2_ for 12–18 h. When the cultured cells reached 80% confluence, HEK293T cells (KCB Cat# KCB 200744YJ) were co-transfected with lentiviral expression constructs pLKO.1-shRNA (4 μg), viral envelope plasmid (pMD2.G, 4 μg), and viral packaging plasmid (psPAX2, 4 μg) using Lipofectamine 3000 (Invitrogen) according to the manufacturer’s protocols. Lentiviral expression constructs with scrambled shRNA were used as controls (specified as Scr). Viral supernatants were collected at 48–72 h post-transfection, filtered through 0.45 μm filters, and stored at −80 °C until use. ISK or KLE cells were seeded and incubated overnight prior to infection. Medium was replaced with a 1:2 dilution of viral supernatant supplemented with 10 μg/mL polybrene, and incubated for 12 h, followed by replacement with normal growth medium. Stable scramble and EZH2 knockdown cell lines were selected using puromycin (2 μg/ml, Sigma-Aldrich) prior to use in experiments. The sequences of shRNAs used in lentivirus expression vector construction for gene knockdown are shown in Supplementary Table S4.

### Western blot analysis

Total protein was extracted from cells using cell lysis buffer for western blots (P0013, Beyotime). Core histones were extracted by an acid-extraction method as previously reported [16]. All proteins were separated by sodium dodecyl sulfate-polyacrylamide gel electrophoresis (SDS-PAGE), followed by semi-dry blotting onto PVDF membranes (Roche). Primary antibodies against EZH2 (Cell Signaling Technology Cat# 5246, 1:1000), p21 (Cell Signaling Technology Cat# 2947, 1:1000), EED (Cell Signaling Technology Cat# 85322, 1:1000), SUZ12 (Cell Signaling Technology Cat# 3737, 1:1000), p53 (Cell Signaling Technology Cat# 2527, 1:1000), E2A (Cell Signaling Technology Cat# 12258, 1:1000), Histone H3 (Abconal Cat# A2348, 1:3000), H3K27me3 (Abcam Cat# ab6002, 1:2000), DNMT1(Abconal Cat# A5495, 1:1000), DNMT3A (Abcam Cat# ab2850, 1:1000), DNMT3B (Abcam Cat# ab2851, 1:1000), β-Tubulin (ABclonal Cat# A12289, 1:1000) and GAPDH (MBL Cat# M171-3MS, 1:3000,) were used. HRP-conjugated goat anti-rabbit IgG (ABclonal Cat# AS014, 1:10000) and goat anti-mouse IgG (ABclonal Cat# AS003, 1:10000) were used as the secondary antibody. The blots were developed using Western ECL Blotting Substrate. Blots were exposed to X-ray film, and the films were scanned with an Epson Perfection V700 Photo Scanner.

### Chromatin immunoprecipitation (ChIP) assay

ChIP assays were performed with ISK cells as described previously [17]. Antibodies used for ChIP were: anti-H3K27me3 (Abcam Cat# ab6002), and anti-H3K27me2 (Sigma-Aldrich Cat# 17-10108). Normal rabbit IgG (Beyotime Cat# A7016) and mouse IgG (Beyotime Cat# A7028) served as controls; the final ChIP DNAs were then used as templates in qPCR reactions, using primers that encompass the promoter region of interest. See Supplementary Table S5 for ChIP-qPCR primer sequences.

### CCK8, colony formation, and EdU assay

For CCK8 assay, cells were seeded in 96-well plates at a concentration of 1 × 10^3^ cells per well. CCK8 assay (Vazyme Biotech) was carried out after incubation for 4 days. The optical density (OD) was read at an absorbance of 450 nm using a multifunction microplate reader (Safire, TECAN). For colony formation assay, cells were seeded in 6-well plates at a concentration of 500–1000 cells per well. Cells were treated with indicated compounds, and culture medium was refreshed every 2 days for about 2 weeks. Colonies were fixed with methanol at room temperature for 30 min, and then stained with crystal violet (0.1% w/v in methanol) for 15 min and photographed. For EdU assay, cells were seeded in 96-well plates at a density of 2 × 10^3^ cells per well, and the assay was carried out according to the instructions for the EdU assay kit (RiboBio). All assays described were performed independently at least three times.

### Cell cycle and apoptosis analysis

Stable EZH2 knockdown (KD1/2) and Scramble (Scr) endometrial adenocarcinoma cells (ISK and KLE) were dissociated with 0.25% trypsin without EDTA and harvested by centrifugation. Cell cycles were detected by Cell Cycle Detection Kit (KeyGen). Briefly, collected cells were washed twice in PBS, and fixed with 500 μL 70% ice-cold ethanol at 4 °C overnight. The fixed cells were washed with PBS before staining. Cells were resuspended in 500 μL PI/RNase A staining solution for 30 min at room temperature in the dark, and analyzed on a BD FACScalibur Flow cytometer. Apoptosis was determined using an Annexin V-FITC/PI Apoptosis Detection Kit (KeyGen). Briefly, collected and washed cells (as above) were resuspended in 500 μL of binding buffer containing 5 μL Annexin V-FITC antibody (Thermo Fisher Scientific Cat# BMS500FI/100) and 5 μL Propidium Iodide and incubated for 10 min at room temperature in the dark. Samples were analyzed on a BD FACScalibur Flow cytometer.

### Gene microarrays

Total RNA was harvested using Trizol reagent (Invitrogen). Hybridization and scanning of the chips (Affymetrix GeneChip PrimeView^TM^ Human Gene Expression Array) was performed as outlined in the Affymetrix technical manual by CapitalBio Corp. (Beijing, China). Differentially expressed genes were identified using Student’s *t*-test for comparison of two groups. The threshold set for up- and downregulated genes was a fold change >2.0 and *P* < 0.05. The microarray data have been deposited in the GEO database (www.ncbi.nlm.nih.gov/geo/) under accession number GSE139246. For gene set enrichment analysis (GSEA), normalized expression data were analyzed, and visualized with the GSEA software (SeqGSEA). The normalized enrichment score (NES) and false discovery rate (FDR) were calculated for comparison.

### TCF3 promoter methylation analysis

Genomic DNA was extracted using the DNeasy Tissue Kit (Qiagen). Bisulfite conversion was performed using the EZ DNA Methylation-Gold^TM^ Kit (Qiagen) and bisulfite genomic sequencing was performed according to the manufacturer’s instructions. The PCR primers used for bisulfite sequencing (BS) are provided in Supplementary Table S6.

### In-vivo tumor models

Animal experiments were performed according to the National Institutes of Health’s Guide for the Care and Use of Laboratory Animals, and were approved by the ethics committee of Jiangsu Provincial Hospital on Integration of Chinese and Western Medicine (Nanjing, China). Cell line-derived xenograft (CDX) and patient-derived xenograft (PDX) mouse models were used in this study. The sample size was chosen with adequate power on the basis of the literature and our previous experience [15], and for each experiment it is indicated in the figure legend. Prior to carrying out the experiment, mice were randomly assigned to different treatment groups.

To establish CDX xenografts, 6-week old female BALB/c nude mice obtained from the Model Animal Research Center of Nanjing University (Nanjing, China) were maintained under defined conditions at the Animal Experiment Center of Nanjing University. Lentiviruses used were EZH2-KD1, EZH2-KD2, TCF3-KD1, TCF3-KD2, and Scr. KLE cells were transduced individually with Scr, EZH2-KD1, or TCF3-KD1, or with a combination of EZH2-KD1 and TCF3-KD1 lentiviruses to establish stable cell lines used for in vivo functional verification studies. Also, parental KLE cells non-transduced with lentivirus was used for in vivo drug studies. Subsequently, 4 × 10^6^ transfected cells in 0.2 μL of RPMI 1640 : Matrigel (1:1, v/v) were injected subcutaneously (s.c.) into the right flank of each mouse (6 mice/group). Tumor size was measured every week with a digital caliper, and volume was calculated using a standard modified formula: Volume (mm^3^) = 0.52 × (length × width^2^).

For the PDX mouse model, the EC PDX studies were conducted by Nanchang Royo Biotech Co., Ltd. This PDX model was derived from a female endometrial adenocarcinoma patient with FIGO stage II undergoing surgery at the People’s Hospital of Jiangxi Province. Informed consent was obtained from the patient, and all procedures involving human samples were approved by the medical ethical committee of the People’s Hospital of Jiangxi Province.

### In-vivo drug studies

Established CDX and PDX models were used for drug treatment studies. Tumor-bearing mice were randomized into groups, and drug dosing was initiated when average tumor volume reached 50–100 mm^3^, which was assigned as day 1 of the treatment regimen. GSK126 dissolved in 20% SBE-β-CD, pH 4.5, was administered i.p. at a dose of 100 mg/kg body mass. 5-Aza dissolved in 1% DMSO and 18% SBE-β-CD was administered i.p. at a dose of 2.5 mg/kg body mass. Tumor growth was monitored by the measurement of subcutaneous tumor volume as above. Mouse body weight was also measured every two days after dosing. All mice were euthanized and tumor tissues collected 8 h after the last dosing. Tumor tissues were weighed and prepared for immunoblotting or immunohistochemical staining. Tumor growth inhibition (TGI) rate was measured using the formula: TGI (%) = [1 − (*V*_t_ − *V*_0_ in treated group)/*V*_t_ − *V*_0_ in vehicle group)] × 100 where *V*_t_ is the volume on each day and *V*_0_ is the volume at the beginning of the treatment.

### Combination treatment and criteria for synergy in vitro

The combination therapies were performed in 96-well plates. Cells were treated with GSK126, 5-Aza, or the combination at graded concentrations for 7 days, and cell growth was measured by CCK-8 assay. To determine the presence of a possible synergistic effect of GSK126 and 5-Aza, the combination index (CI) was calculated by CompuSyn Version 1.0 software. A CI < 0.8 indicates synergy and CI > 1.2 indicates an antagonistic effect.

### Statistical analysis

Data analysis was performed with the statistical program GraphPad Prism (GraphPad Prism). Results are presented as mean ± SD unless otherwise indicated. Statistical analyses were performed using two-tailed Student’s *t*-test to derive the significance of the differences between the two groups. *P* < 0.05 was considered to be significant.

## Results

### Upregulation of EZH2 correlates with poor prognosis in EC, and promotes EC cell proliferation in vitro

**Fig. 1:**
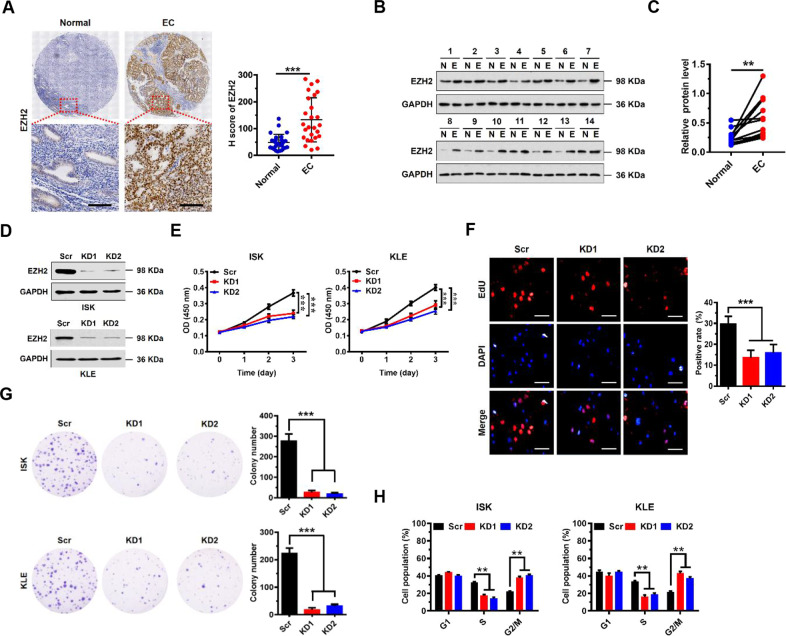
Upregulation of EZH2 correlates with poor prognosis in EC, and promotes EC cell proliferation in vitro. **A** Immunohistochemical (IHC) staining of EZH2 protein in EC and matched human normal tissues (as indicated). Representative micrographs are shown in original magnification (×400). Scale bars, 50 μm. Total IHC score of EZH2 in EC and matched human normal tissues (*n* = 26, right). ***P < 0.001 compared to the Normal control. **B** Western blot analysis of EZH2 in cell lysates from normal (N) and EC tumor (E) tissues (*n* = 14). GAPDH served as a loading control. **C** EZH2 protein expression levels by quantitation of density of protein bands from western blot in EC tissues in (**B**) relative to the normal tissues (*n* = 14, **P < 0.01). **D** Western blot assay of EZH2 protein expression following EZH2 knockdown in ISK and KLE cells. GAPDH served as a loading control. **E** Proliferation of EC cells transfected with siRNA to EZH2 (EZH2 knockdown, KD1 or KD2) or scramble control (Scr) in both ISK and KLE cells. Values at the indicated time points represent mean ± SD from three independent tests; ***P < 0.001 vs. Scr control. **F** EdU proliferation analysis of the effect of siRNA KD of EZH2 on the growth of ISK cells compared to Scr controls. Scale bars, 25 μm; ***P < 0.001 vs. Scr control. **G** Colony formation assay of ISK cells with knockdown of EZH2 or scramble control; ***P < 0.001 vs. Scr control. **H** The percentages of ISK and KLE Scr or EZH2 knockdown cells in the G_1_, S, and G_2_/M phases analyzed by flow cytometry showing cell cycle distribution. Each histogram bar represents the mean ± SD of three independent experiments. **P < 0.01 vs. Scr control.

To investigate the clinical relevance of EHZ2 expression in patients with EC, we first examined EZH2 expression in EC tissue specimens from a cohort of 26 EC patients by immunohistochemistry using a specific anti-EZH2 antibody (Supplementary Table S1). Significantly higher expression levels of EZH2 were observed in EC tissues compared with matched adjacent normal tissues, and EZH2 was predominantly localized in the nuclei of glandular epithelium cells (Fig. 1A and Supplementary Fig. S1A). Western blot analysis confirmed that the expression levels of EZH2 in the tumor tissues were significantly higher than in matched adjacent normal tissues (Fig. 1B, C). Notably, EZH2 expression correlated with increased tumor invasion and higher grade, but not with lymph node status (Supplementary Table S1). These results are consistent with meta-analysis of transcriptome data extracted from The Cancer Genome Atlas (TCGA) Data Portal which show that EZH2 mRNA levels in EC patient tumor tissues were significantly higher than adjacent normal tissues (Supplementary Fig. S1B). Importantly, EC patients with high EZH2 expression had shorter overall survival (Supplementary Fig. S1C). These results indicate that EZH2 protein levels were upregulated in EC tissues, and that high levels of EZH2 expression correlate with poor prognosis in EC.

To assess the potential role of EZH2 in EC development, we evaluated the effect of EZH2 on cell proliferation, cell cycle, and apoptosis. We generated two independent, stable EZH2 knockdowns (EZH2 KD1 and KD2) in both human ISK and KLE EC cell lines using lentiviral vectors containing different specific shRNAs targeting EZH2 mRNA. The knockdown efficiency of EZH2 was confirmed by Western blot analysis (Fig. 1D) and quantitative RT-PCR (Supplementary Fig. S2A). Knockdown of EZH2 significantly reduced the cell growth rate of both ISK and KLE cell lines (Fig. 1E) compared with scrambled control (Scr) cells. These results were further confirmed by EdU staining to detect nucleotide analogue incorporation into replicated DNA (Fig. 1F). Consistently, stable knockdown of EZH2 significantly reduced the number of cell colonies formed after culture compared with Scr cells (Fig. 1G). Further, we performed flow cytometric analysis of the cellular DNA content of both ISK and KLE cells to assess the effect of EZH2 on cell cycle progression. We observed more EZH2-KD cells in G2/M phase than Scr cells, and correspondingly, fewer EZH2-KD cells in the S phases of the cell cycle (Fig. 1H). However, no differences in numbers of apoptotic cells were found between knockdown and Scr cells (Supplementary Fig. S2B). All together, these results indicate that knockdown of EZH2 induces G2/M cell cycle arrest in EC cells, resulting in inhibition of proliferation of EC cells.

### CCKN1A (p21^WAF1/Cip1^) is an indirect downstream target of EZH2

**Fig. 2:**
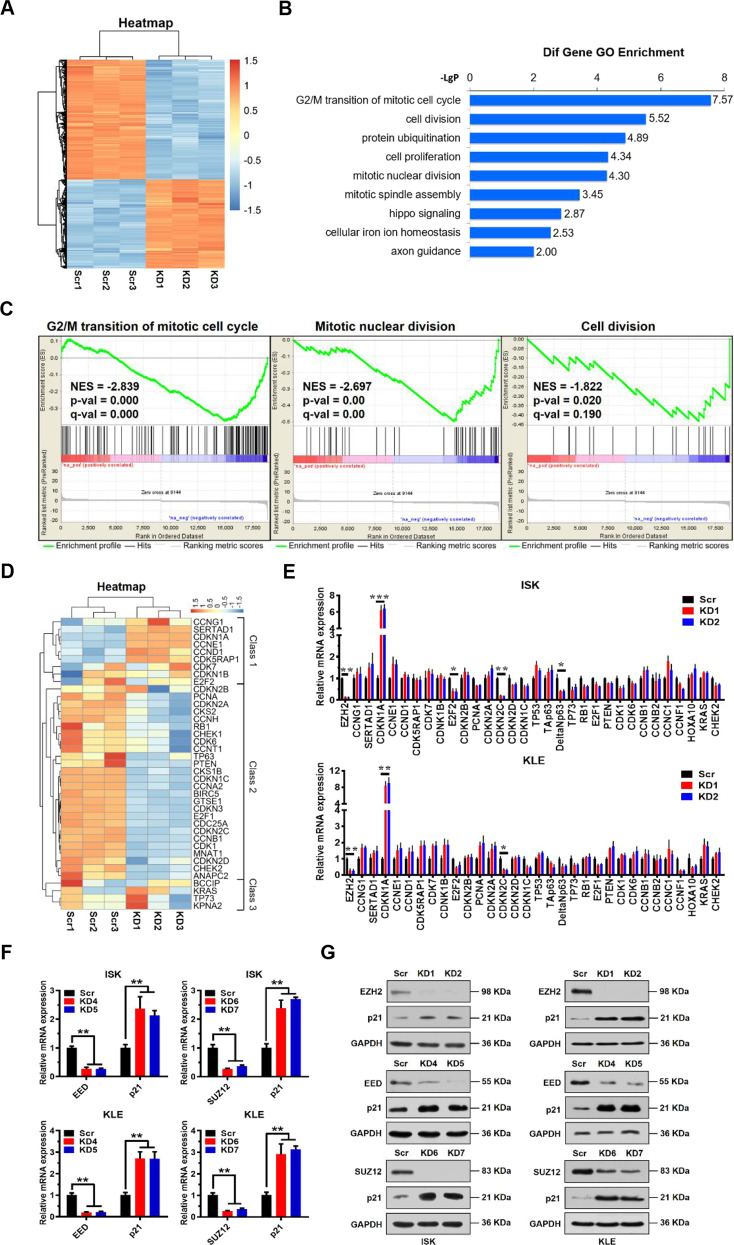
Identification of p21 as an indirect target of EZH2. **A** Hierarchical cluster analysis of all genes exhibiting differential expression in EZH2 knockdown (KD 1, 2, 3) or Scr control (Scr 1, 2, 3) ISK cells. **B** Significant biological processes of differentially expressed genes (both up and down) analyzed by Gene Ontology (GO) enrichment. The *Y* axis is the pathway category, and the *X* axis is the negative logarithm of the *P* value (−LgP). **C** Gene set enrichment analysis (GSEA) for G_2_/M transition, mitotic nuclear division, or cell division of ISK cells with or without EZH2 knockdown; *n* = 3 per group. **D** Hierarchical cluster analysis of key genes related to cell cycle regulation in EZH2 knockdown (KD) or Scr control ISK cells. **E** Relative mRNA expression levels of a panel of key genes involved in cell cycle regulation analyzed by quantitative real-time PCR in Scr control or EZH2 knockdown ISK and KLE cells. GAPDH was used as an endogenous control. Data shown are mean ± SD (*n* = 3). ***P < 0.001, **P < 0.01, and *P < 0.05 vs. Scr control. **F** Relative mRNA expression levels of p21 normalized to GAPDH analyzed by quantitative real-time PCR in Scr control, EED knockdown (KD 4, 5) or SUZ12 knockdown (KD 6, 7) ISK and KLE cells. Data shown are mean ± SD (*n* = 3). ***P < 0.001, **P < 0.01 and *P < 0.05 vs. Scr control. **G** Immunoblots of indicated proteins in Scr, EZH2 knockdown, EED knockdown, or SUZ12 knockdown ISK and KLE cells. GAPDH served as a loading control.

To further investigate how and why EZH2 impacts proliferation of EC cells, we performed microarray gene expression profiling analysis using ISK cells without (Scr 1, 2, 3) or with EZH2 knockdown (KD 1, 2, 3). We identified 1819 upregulated and 2450 downregulated mRNAs in EZH2 knockdown cells compared to Scr cells (Fig. 2A, GEO database under accession number GSE139246). The effect of EZH2 knockdown on gene function categories was analyzed by GO enrichment analysis. The most significant differentially expressed category of genes related to biological function was G2/M transition of mitotic cell cycle, which may include important cell cycle checkpoint genes (Fig. 2B). Gene set enrichment analysis (GSEA) indicated that EZH2 knockdown differentially affected the expression of genes associated with G2/M transition, mitotic nuclear division, and cell division (Fig. 2C). Collectively, these results indicate that EZH2 function is tightly associated with cell cycle transition.

Subsequently, we performed a heatmap analysis of differentially expressed genes related to cell cycle regulation. Cluster analysis of heatmap data indicated that 39 differentially expressed genes could be grouped into 3 classes (Fig. 2D). EZH2 mediates H3K27me3 modification, which is associated with chromatin compaction and gene silencing. Thus, EZH2 knockdown decreases the overall level of H3K27me3, thereby activating gene expression. On the heatmap, we found that one class of genes including CCNG1, SERTAD1, CDKN1A, CCNE1, CCND1, and CDK5RAP1, which play key roles in cell cycle regulation and are involved in G2/M cell cycle transition, were significantly upregulated when EZH2 was knocked down (Fig. 2D). Therefore, we analyzed expression of these genes and some other cell cycle-associated genes by qRT-PCR. We found that CDKN1A (also known as p21^WAF1/Cip1^) expression was the most upregulated gene in both ISK and KLE EZH2-KD cell lines compared to Scr cells (Fig. 2E). Since the PRC2 complex has two other core subunits in addition to EZH2, EED, and SUZ12, we knocked down EED (KD4, KD5) and SUZ12 (KD6, KD7), as well, to assess their effect on p21 expression in both ISK and KLE cells. As expected, we observed marked upregulation of p21 expression at both mRNA and protein levels in these cells (Fig. 2F, G), supporting a role of EZH2 in repressing expression of p21. However, ChIP results indicated that there was no significant EZH2-mediated enrichment of H3K27me3 or H3K27me2 on the p21 promoter (Supplementary Fig. S3A), suggesting that EZH2 may not regulate p21 gene expression directly. Thus, these results indicated that EZH2 participates in cell cycle regulation in EC cells, probably through indirect suppression of p21 expression.

### TCF3 is a direct target of EZH2 which acts as a tumor suppressor in EC cells

**Fig. 3:**
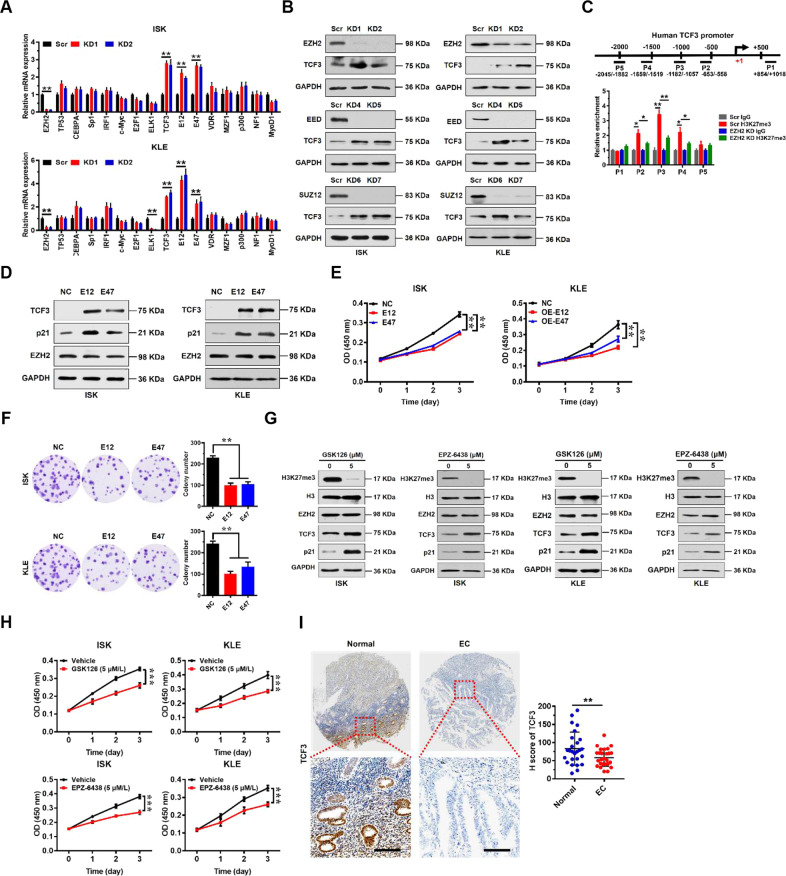
EZH2 directly regulates TCF3 which regulates p21 transcription in EC cells. **A** Relative mRNA expression levels of a panel of key transcription factors potentially regulating p21 normalized to GAPDH analyzed by quantitative real-time PCR in scrambled control (Scr) or EZH2 knockdown ISK and KLE cells. Data shown are mean ± SD (n = 3). **P < 0.01 compared with Scr control. **B** Immunoblots of EZH2 and TCF3 in Scr or PRC2 core subunit (EZH2, EED, and SUZ12) knockdown ISK and KLE cells. GAPDH served as a loading control. **C** Enrichment of H3K27me3 on TCF3 promoter in Scr or EZH2 knockdown ISK cells detected by ChIP-qPCR (*bottom*). Diagram shows positions of ChIP primers at the TCF3 promoter (top). Data shown are mean ± SD (*n* = 3). **P < 0.01, *P < 0.05 compared with indicated control. **D** Immunoblots of TCF3, p21, and EZH2 in negative control (NC, vector alone) or enforced expression of TCF3 variants (E12 or E47) in ISK and KLE cells. GAPDH served as a loading control. **E**, **F** Cellular proliferation were measured by CCK8 (**E**) and colony formation assay (**F**) after enforced expression TCF3 variants (E12 or E47) in ISK and KLE cells. **P < 0.01 vs. NC. **G** Effect of inhibition of EZH2 activity by GSK126 and EPZ-6438 on TCF3 and p21 protein levels in ISK (left) and KLE (right) cells. GAPDH served as a loading control. **H** Cell proliferation measured by CCK8 assay after GSK126 and EPZ-6438 treatment in ISK and KLE cells. **P < 0.01 vs. Vehicle treatment. **I** Immunohistochemical (IHC) staining of TCF3 protein in matched human normal and EC tissues (left). Representative micrographs are shown in original magnification (×400). Scale bars, 50 μm. Total IHC score of TCF3 in matched human normal and EC tissues (*n* = 26). ***P < 0.001 vs. Normal control (right).

Given that EZH2 might regulate, indirectly, transcription of p21, we reasoned that there was a transcription factor or other molecule that were regulated by EZH2 directly to bridge the gap between EZH2 and p21. Therefore, we examined the sequence of the p21 promoter. According to PROMO database and previous reports, we found that the p21 promoter had potential binding sites for a number of transcription factors (TF): TP53, CEBPA, Sp1, IRF1, c-Myc, E2F1, ELK1, TCF3, VDR, MZF1, p300, NF1, and MyoD1 (Supplementary Table S2). We then assessed changes in expression levels of these TFs in EZH2 knockdown cells using qRT-PCR. We found that TCF3 (also called E2A) and its two splice variants (E12 and E47) mRNA levels were significantly upregulated in EZH2 knockdown ISK and KLE cells compared with Scr cells (Fig. 3A). Notably, in our examination of the other potential transcription factors, we found no significant upregulation of p53 expression in two EZH2 KD cell lines (Fig. 3A and Supplementary Fig. S3B). Furthermore, knockdown of the other PRC2 subunits, EED and SUZ12, also upregulated TCF3 expression (Fig. 3B). Conversely, we found that overexpression of EZH2 downregulated TCF3 and p21 expression (Supplementary Fig. S3C). Therefore, we checked EZH2-mediated H3K27me3 enrichment on the TCF3 promoter. We identified H3K27me3 enrichment peaks on the TCF3 promoter upstream of transcription start site (−1182/−1057) (Fig. 3C). As expected, knockdown of EZH2 reduced cellular levels of H3K27me3 and its enrichment on the TCF3 promoter accordingly (Fig. 3C and Supplementary Fig. S3D).

TCF3 is known to have two splice variant products, E12 and E47 [18], which can act as either oncoproteins or tumor suppressors in a context-dependent manner. Because there was low expression of TCF3 in EC cells, we examined the effect of forced expression of the two splice variants of TCF3, E12, and E47, on EC proliferation rather than attempting to knockdown E12 or E47 in ISK and KLE cells (Fig. 3D). We found that p21 expression was significantly increased upon overexpression of E12 or E47 but no change in EZH2 expression levels (Fig. 3D). We also found that overexpression of the splice variants significantly inhibited proliferation and colony formation of ISK and KLE cells (Fig. 3E, F).

To further test the possibility that EZH2 methyltransferase activity was essential for TCF3 regulation and EC cell proliferation, we examined TCF3 expression levels and cell proliferation in the presence of EZH2-specific small molecular inhibitors (GSK126 and EPZ-6438). We found that TCF3 expression was indeed upregulated by in the presence of EZH2 inhibitors, which was accompanied by decreased H3K27me3 levels (Fig. 3G). Of note, we found that p21 upregulation upon GSK126 or EPZ-6438 treatment was much more evident relative to TCF3 upregulation (Fig. 3G), suggesting that some other mechanisms in addition to TCF3 transcription factor may also be involved in EZH2-mediated p21 repression. Proliferation of ISK and KLE cells was significantly inhibited by GSK126 and EPZ-6438 (Fig. 3H). These results indicate that TCF3 could be a direct target of EZH2, and that methyltransferase activity is key for TCF3 expression and for proliferative capacity of EC cells.

To assess the clinical relevance of TCF3 expression in patients with EC, we examined TCF3 expression in EC tissue specimens from a cohort of 26 EC patients by immunohistochemistry using a specific anti-TCF3 antibody. Tissue array analysis showed that TCF3 was mainly localized to glandular epithelium, and its expression levels were markedly decreased in the EC tissues compared to the adjacent normal tissues (Fig. 3I). These results are consistent with meta-analysis of transcriptome data extracted from The Cancer Genome Atlas (TCGA) Data Portal, which show that EC patients with lower TCF3 expression had shorter overall survival (Supplementary Fig. S3E). These results indicate that TCF3 protein levels were downregulated in EC tissues, and that low levels of TCF3 expression correlate with poor prognosis in EC.

Taken together, these results demonstrate that TCF3 is a direct target of EZH2, which may act as a tumor suppressor to inhibit proliferation of EC cells.

### EZH2/TCF3/p21 axis promotes EC cell proliferation

**Fig. 4:**
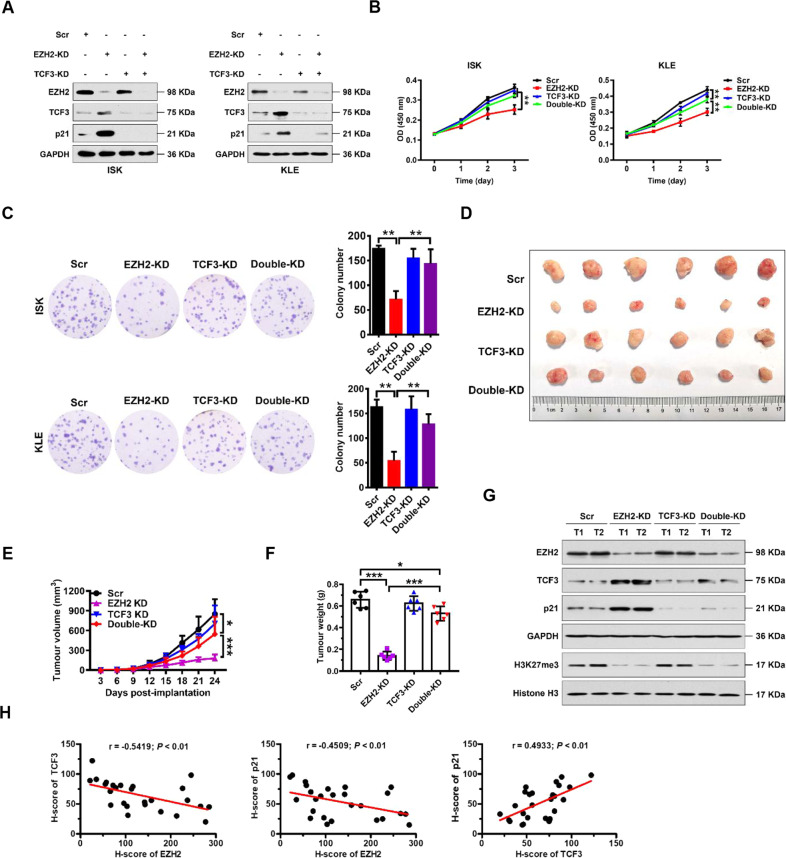
EZH2 promotes proliferation of EC cells by directly repressing TCF3. **A** Immunoblots of EZH2, TCF3, or p21 following knocking down of EZH2 or TCF3 individually or simultaneously in ISK (left) and KLE (right) cells. GAPDH was used as internal loading control. **B**, **C** Cellular proliferation measured by CCK8 (**B**) and colony formation (**C**) assays following knockdown of EZH2 or TCF3 individually or simultaneously in ISK and KLE cells (*as indicated*). **P < 0.01 vs. indicated control. **D**–**F** Photograph (**D**), tumor volumes (**E**), and tumor masses (**F**) of excised xenograft tumors from scrambled (Scr) control, EZH2-KD, TCF3-KD, and EZH2-KD + TCF3-KD groups. Data are presented as the means ± SD; ***P < 0.001, *P < 0.05 vs. indicated control. **G** Western blotting analysis of indicated proteins in excised xenograft tumors from Scr control, EZH2-KD, TCF3-KD, and EZH2-KD + TCF3-KD groups. GAPDH and histone H3 were used as endogenous loading controls. **H** Pearson correlation scatter plot of H scores (protein levels) of EZH2 and TCF3 (left) or p21 (middle), or H scores (protein levels) of TCF3 and p21 (right) in human EC (*n* = 26).

To determine whether repression of p21 by EZH2 was dependent on TCF3, we established stable EC cell lines in which EZH2 was knocked down (EZH2-KD), TCF3 was knocked down (TCF3-KD), or both were knocked down. We first examined the effect of TCF3 and EZH2 knockdown on p21 expression by qRT-PCR and western blot analysis (Fig. 4A and Supplementary Fig. S4A). We found that p21 expression was upregulated by knocking down EZH2, and was almost silenced by knocking down TCF3 in both ISK and KLE cells (Fig. 4A). Interestingly, we observed that upregulation of p21 expression by knocking down EZH2 was blocked by simultaneous knockdown of TCF3, suggesting that TCF3 may be a key bridge, linking regulation of EZH2 and p21 in EC cells (Fig. 4A and Supplementary Fig. S4A). TCF3 knockdown also restored the proliferative and colony formation defects exhibited by EZH2 knockdown cells (Fig. 4B, C). In addition, we observed that downregulation of p21 expression by ectopically overexpressing EZH2 was reversed by simultaneously overexpressing TCF3 in EC cells (Supplementary Fig. S8A, B). TCF3 overexpression also reversed the proliferative and colony formation effects exhibited by EZH2-overexpressing cells (Supplementary Fig. S8C, D). These results provide further evidence that TCF3 is a key regulator of p21 expression under the control of EZH2 in vitro.

To further characterize this putative EZH2/TCF3/p21 regulatory axis in vivo, we exploited a subcutaneous xenograft tumor model using KLE cells with EZH2 knockdown or TCF3 knockdown individually or simultaneously. In line with the in vitro results, we observed that knockdown of EZH2 alone significantly inhibited tumor growth. However, the inhibitory effect by EZH2 knockdown was largely abrogated by TCF3 knockdown (Fig. 4D–F). Of note, there were no significant differences in the weight of mice at any time between experimental groups (Supplementary Fig. S4B). The intratumoral levels of EZH2, H3K27me3, TCF3, p21, and Ki67 were analyzed by western blot and immunohistochemical staining (IHC) (Fig. 4G and Supplementary Fig. S4C). Interestingly, the expression levels of EZH2 correlated inversely with levels of TCF3 and p21, and levels of TCF3 and p21 correlated positively in human EC tissues (Fig. 4H). These results indicate that in the EZH2/TCF3/p21 axis, EZH2 is a negative regulator of TCF3 and of p21, and TCF3 is a positive regulator of p21 in vitro and in vivo.

### DNMT3B mediates TCF3 promoter methylation independently of EZH2

**Fig. 5:**
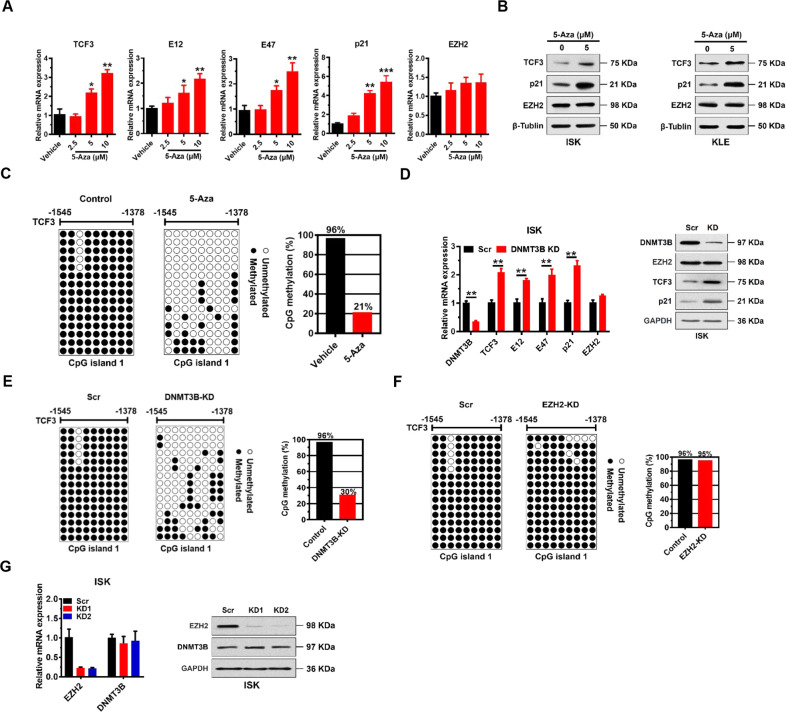
DNMT3B mediates TCF3 promoter methylation independently of EZH2. **A** Relative mRNA expression levels of indicated proteins normalized to GAPDH analyzed by quantitative real-time PCR in KLE cells treated with 5-Aza for 7 days. Data shown are mean ± SD (*n* = 3). *P < 0.05, **P < 0.01, ***P < 0.001 vs. vehicle treatment. **B** Immunoblots of TCF3, p21, and EZH2 in ISK and KLE lysates after 5 μM 5-Aza treatment for 7 days. β-Tubulin served as a loading control. **C** Bisulfite sequencing analysis of the TCF3 promoter (CpG island 1) in KLE cells treated with vehicle control or 5 μM 5-Aza for 4 days. **D** qRT-PCR (left) and immunoblots (right) of indicated proteins in ISK cells after DNMT3B knockdown. **P < 0.01 vs. Scr control. **E** Bisulfite sequencing analysis of the TCF3 promoter (CpG island 1) in ISK cells after DNMT3B knockdown. Each row shows the methylation status of individual CpG dinucleotides derived from sequence analysis of 15 representative individual cloned polymerase chain reaction (PCR) products of CpG island 1 after bisulfite modification. The numbers at the top represent the positions of the CpG dinucleotides relative to the transcriptional start site (TSS) of the TCF3 gene. **F** Bisulfite sequencing analysis of the TCF3 promoter (CpG island 1) in ISK cells after EZH2 knockdown as in (**E**). **G** qRT-PCR (left) and immunoblot (right) analysis showing DNMT3B mRNA and protein levels in ISK cells after EZH2 knockdown. GAPDH was used as a loading control.

DNA methyltransferases (DNMTs), which methylate DNA at CpG islands, play important roles in modulating gene expression during tumorigenesis. Intriguingly, when we examined the human TCF3 promoter region (~2.5 kb upstream of the TSS) using MethPrimer software [19], three potential CpG islands were identified: Island 1: −1545 bp ~ −1378 bp; Island 2: −1218 bp ~ −1072 bp; Island 3: −1032 bp ~ −413 bp (Supplementary Fig. S5A). Therefore, we assessed possible methylation at these islands using the DNA methylation inhibitor 5-Aza-2’-deoxycytidine (5-Aza) to test involvement of DNA methylation in the regulation of expression of TCF3, p21, and EZH2. We found that TCF3 expression, and expression of its two variants (E12 and E47) could be upregulated in a dose-dependent manner by 5-Aza treatment KLE cells, suggesting that DNA methylation regulates TCF3 expression in EC cells (Fig. 5A, B). In addition, p21 expression was also activated by 5-Aza treatment, whereas EZH2 expression was unaffected by 5-Aza (Fig. 5A, B). Similar effects were observed in ISK cells (Fig. 5B). We did not observe changes in global cellular levels of H3K27me3 nor enrichment of H3K27me3 on the TCF3 promoter (Supplementary Fig. S5B, C). We found that 5-Aza treatment significantly inhibited proliferation of ISK and KLE cells (Supplementary Fig. S5D).

To dissect TCF3 promoter methylation status, we isolated DNA from KLE cells, and performed bisulfite-sequencing PCR (BSP) assays. BSP results indicated that CpG Island 1 was heavily methylated (96.3%), while Island 2 and Island 3 were hypomethylated (10.7% and ~0%, respectively) in normal KLE cells (Supplementary Fig. S5E). Thus, CpG Island 1 seemed to be the key methylation site. In this regard, we found that methylation levels of CpG Island 1 were significantly reduced by 5-Aza treatment (20.7%) compared to vehicle treatment (96.3%) (Fig. 5C).

There are three main DNMTs: DNMT1, DNMT3A, and DNMT3B that mediate DNA methylation in cells. To identify the methyltransferase responsible for methylation of the TCF3 promoter in KLE cells, we knocked down DNMT1, DNMT3A, and DNMT3B individually, and tested the effect on TCF3 and p21 expression (Supplementary Fig. S5F-G). We found that DNMT3B knockdown significantly upregulated TCF3, E12, E47, and p21 mRNA and protein expression (Fig. 5D), whereas no significant changes in expression of TCF3 or p21 were observed in DNMT1 and DNMT3A knockdown cells (Supplementary Fig. S5F, G).

To further confirm that DNMT3B directly controls methylation the TCF3 promoter, we performed BSP analysis of CpG island 1 DNA of the TCF3 promoter when DNMT3B was knocked down. We found that methylation of CpG island 1 of the TCF3 promoter was reduced to 30% in DNMT3B knockdown cells compared to 96.3% in Scr cells (Fig. 5E). In contrast, we observed no change in DNA methylation of CpG island 1 of the TCF3 promoter in EZH2 knockdown cells (Fig. 5F). Of note, EZH2 expression was not affected by DNMT3B knockdown (Fig. 5D), and DNMT3B expression was not affected by EZH2 knockdown (Fig. 5G). These results indicate that DNMT3B mediates methylation of the TCF3 promoter, and does so in an EZH2-independent manner.

### DNMT3B/TCF3/p21 axis promotes EC cell proliferation

We further evaluated the role of DNMT3B in EC cell proliferation by knocking down DNMT3B. We found that DNMT3B knockdown significantly inhibited proliferation and colony formation of ISK and KLE cells (Supplementary Fig. S6A, B). Furthermore, we found that TCF3 knockdown significantly blocked p21 upregulation by DNMT3B knockdown in both ISK and KLE cells (Supplementary Fig. S6C, D). We observed the same effect of TCF3 knockdown on rescuing cell growth and colony formation defects in DNMT3B cells (Supplementary Fig. S6E, F). In addition, we observed that downregulation of p21 expression by ectopically overexpressing DNMT3B was reversed by simultaneously overexpressing TCF3 in EC cells (Supplementary Fig. S8E, F). TCF3 overexpression also reversed the proliferative and colony formation effects exhibited by DNMT3B-overexpressing cells (Supplementary Fig. S8G, H). Thus, these results demonstrate that DNMT3B may promote EC cell proliferation by repressing TCF3 expression in vitro.

To further investigate the clinical relevance of DNMT3B expression in patients with EC, we examined DNMT3B expression in EC tissue specimens from a cohort of 26 EC patients by immunohistochemistry using a specific anti-DNMT3B antibody (Supplementary Table S1). Significantly higher expression levels of DNMT3B were observed in EC tissues compared with matched adjacent normal tissues from EC patients, and DNMT3B was predominantly localized in the nuclei of glandular epithelial cells (Supplementary Fig. S6G). Notably, DNMT3B expression correlated with increased tumor invasion and higher grade, but not with lymph node status (Supplementary Table S1). These results are consistent with meta-analysis of transcriptome data extracted from The Cancer Genome Atlas (TCGA) Data Portal, which show that DNMT3B mRNA levels in EC patient tumor tissues were significantly higher than adjacent normal tissues (Supplementary Fig. S6H). Importantly, EC patients with high DNMT3B expression had shorter overall survival (Supplementary Fig. S6I). Interestingly, expression levels of DNMT3B correlated inversely with expression of TCF3 and p21 in EC tissues (Supplementary Fig. S6J). These results indicate that DNMT3B protein levels were upregulated in EC tissues, and suggest that high levels of DNMT3B expression may correlate with poor prognosis in EC.

### GSK126 and 5-Aza act synergistically in EC

**Fig. 6:**
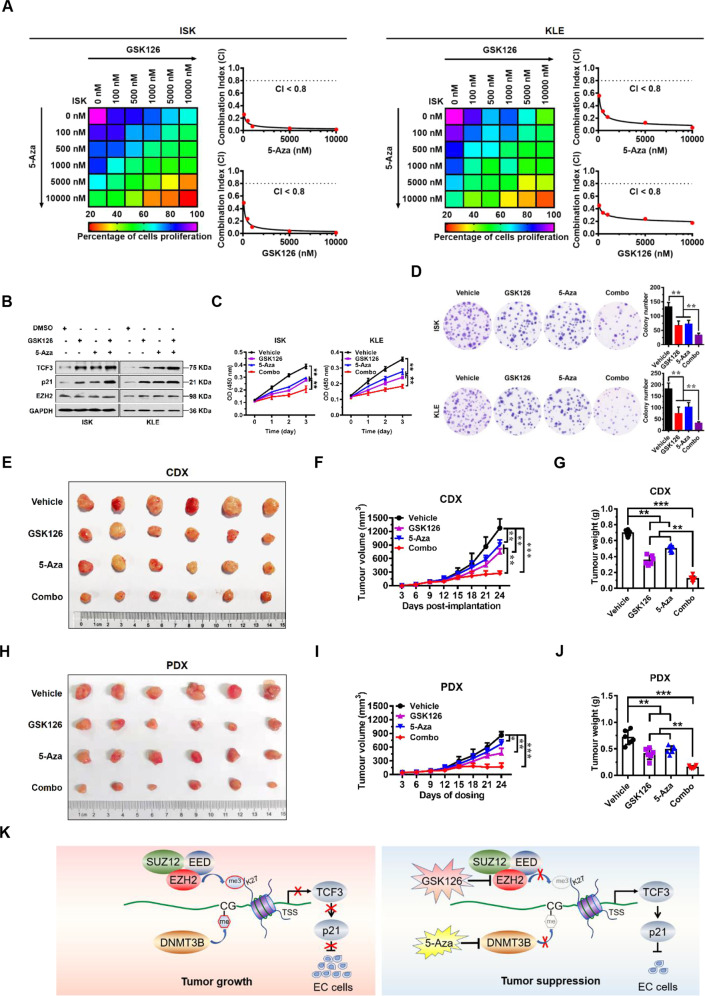
Synergistic effect of combined treatment with GSK126 and 5-Aza in human EC. **A** Drug dose-response matrix for growth inhibition of ISK (left) and KLE (right) cells following 7-day culture with GSK126 plus 5-Aza. Color gradation indicates percentage viability at the indicated dose combination. Combination index (CI) plots for 5 μM 5-Aza (top) or GSK126 (bottom) with graded doses of 5-Aza or GSK126 in ISK and KLE cells. **B** Immunoblots of TCF3, p21, and EZH2 protein in ISK (left) and KLE (right) cells following treatment with GSK126 (5 μM) or 5-Aza (5 μM), or combination for 7 days. GAPDH was used as a loading control. **C**, **D** CCK8 (**C**) and colony formation (**D**) assay were performed to evaluate the effect of the combination treatment with GSK126 and 5-Aza on cell proliferation of ISK and KLE cells. **P < 0.01 vs. indicated control. **E**–**G** Photograph (**E**), tumor volumes (**F**), and tumor masses (**G**) of ISK cell-derived xenograft tumors excised from mice treated with GSK126 (100 mg/kg) or 5-Aza (2.5 mg/kg) or combination. Data are presented as the means ± SD; **P < 0.01, ***P < 0.001 vs. indicated control. **H**–**J** Photograph (**H**), tumor volumes (**I**), and tumor masses (**J**) of patient-derived xenograft tumors excised from mice treated with GSK126 (100 mg/kg) or 5-Aza (2.5 mg/kg) or combination. Data are presented as the means ± SD; *P < 0.05, **P < 0.01, ***P < 0.001 vs. vehicle control. **K** Model of EC progression driven by PRC2 complex (EZH2, EED, and SUZ12) and DNMT3B. PRC2 complex and DNMT3B epigenetically repress transcription of TCF3 and p21, and promote EC cell proliferation (left). Inhibition of EZH2 and DNMT3B by GSK126 and 5-Aza de-represses TCF3 and p21 expression, and blocks EC tumor growth (right).

Given that EZH2 and DNMT3B promote EC cell proliferation through epigenetic activation of TCF3, we sought to test whether combined inhibition of EZH2 and DNMT3B would exert a better therapeutic effect in EC. To this end, we initially examined the effect of a combination of GSK126 and 5-Aza on cell proliferation. Heatmap and Combination index (CI) [20] plots of combination treatment of GSK126 with 5-Aza showed that the two reagents synergistically improved the anti-proliferative effect on EC cells (CI < 0.8, Fig. 6A). Based on these results, designated concentrations of GSK126 (5 μM) and 5-Aza (5 μM) were used in further cellular studies. Results of western blot assays demonstrated that the selected combination of GSK126 and 5-Aza achieved robust activation of TCF3 and p21 expression (Fig. 6B). Combinational treatment with GSK126 and 5-Aza profoundly blocked proliferation and colony formation capacity of EC cells (Fig. 6C, D). Similar synergistic results were obtained from the combinational treatment with EPZ-6438 and 5-Aza on EC cells (Supplementary Fig. S9A–D).

To confirm these results in vivo, we utilized both a cell line-derived xenograft (CDX) model and a primary endometrioid adenocancer patient-derived xenograft (PDX) model to test the therapeutic benefits of the EZH2/DNMT3B combo-inhibitor. In the CDX model, daily single treatment with GSK126 (100 mg/kg) or 5-Aza (2.5 mg/kg) had a moderate therapeutic effect, with a tumor growth inhibition (TGI) rate of 41.3% or 29.0%, respectively (Fig. 6E–G). Consistent with the previous in vitro findings, EZH2/DMMT3B combo-inhibitor (GSK126 + 5-Aza) greatly inhibited tumor growth, showing a TGI rate of about 78.7% (Fig. 6E–G), but had no effect on mouse body weight (Supplementary Fig. S7A). These results indicate that the combined intervention had a synergistic inhibitory effect on subcutaneous EC tumor growth. Correspondingly, we also detected higher protein levels of TCF3 and p21 in the combination-treatment group than in the single-treatment groups by western blot and IHC analysis (Supplementary Fig. S7B, C).

Next, we further tested the benefit of the EZH2/DNMT3B combo-inhibitor in the PDX model. We found that daily single treatment with 5-Aza at 2.5 mg/kg had a marginal therapeutic effect, with a TGI rate of 21.7%, and GSK126 at 100 mg/kg had a TGI rate of 45.8%. In contrast, the combination treatment with GSK126 and 5-Aza displayed robust efficacy with very tolerable toxicity, showing a TGI rate of about 80.2% (Fig. 6H–J and Supplementary Fig. S7D), suggesting a synergistic inhibitory effect on PDX EC tumor growth. Consistently, we detected higher protein levels of TCF3 and p21 in the combination-treatment group than in the GSK126- or 5-Aza-single-treatment groups by western blot and IHC analysis (Supplementary Fig. S7E, F). Similar synergistic inhibitory results were obtained from the combinational treatment with EPZ-6438 and 5-Aza on CDX EC tumor growth (Supplementary Fig. S10A–E) and PDX EC tumor growth (Supplementary Fig. S10F–J). Taken together, these data demonstrate that in combination GSK126 and 5-Aza exert profound drug synergy in EC.

## Discussion

In this study, we demonstrated that TCF3 is epigenetically silenced by EZH2 and DNMT3B and functions as a tumor suppressor in endometrial cancer. TCF3 (also called E2A) belongs to the family of basic helix loop helix (bHLH) proteins, and encodes two alternatively spliced variants E12 and E47 [18]. TCF3 proteins form homodimers or heterodimers with other bHLH proteins to conduct their tissue- or cell type-specific functions [21]. TCF3 protein was initially reported to play particularly important roles during lymphocyte development [22], and it is dysregulated in various cancers including lymphoma, pancreatic cancer, breast cancer, colorectal cancer, and prostate cancer [23–27]. Considerable studies had shown that TCF3 protein can act as either a tumor suppressor [27] or an oncoprotein [23]. Thus, there is some ambiguity of the role of TCF3 in cancer development. In the current study, we identified TCF3 as a direct target of EZH2 and DNMT3B, which acts as a tumor suppressor in EC. More importantly, TCF3 expression levels are positively associated with prognosis in EC patients, and inversely correlate well with EZH2 and DNMT3B expression levels in EC samples. Our results, therefore, suggest a potential prognostic value of TCF3 as a biomarker for EC.

Relationships between DNA methylation and histone modification have implications for understanding normal development as well as tumorigenesis [28]. Individual epigenetic modifications, including H3K27me3 and DNA methylation, have physiological functions in normal cells, and cancer cell specificity is hard to achieve therapeutically using a drug that targets a single epigenetic modification [29]. Recent studies indicate that dual inhibition of DNMTs and EZH2 exhibits synergistic antineoplastic activity against human myeloid leukemia cells [30], and can resensitize the resistant myeloma cells to both lenalidomide and pomalidomide in vitro [31]. These data highlight the need to target multiple epigenetic abnormalities through the combined use of an EZH2 inhibitor and 5-Aza. Our results may provide a rational for developing a new therapeutic approach for EC.

In summary, our findings show that the combined application of GSK126 and 5-Aza synergistically inhibited EZH2 and DNMT3B to derepress the expression of TCF3, and profoundly blocked EC tumor progression in mouse models (Fig. 6K). These findings support the notion that targeting the EZH2/DNMT3B/TCF3/p21 axis may be a novel therapeutic strategy to further improve the efficacy of EC treatment.

## Supplementary information

Supplemental figure legends

Supplemental figures and tables

## Data Availability

Our gene-microarray sequencing data have been deposited in the Gene Expression Omnibus (GEO) under accession number GSE139246.
