# Supplementary material for: TCF3 is epigenetically silenced by EZH2 and DNMT3B and functions as a tumor suppressor in endometrial cancer
Source: Cell Death Differ. 2021 Jun 26;28(12):3316–28. doi: 10.1038/s41418-021-00824-w (PMC8630057; doi:10.1038/s41418-021-00824-w)
Supplement: Supplementary file 1 — Supplemental figure legends [file 41418_2021_824_MOESM1_ESM.doc]

**Supplementary figure legends**

Fig. S1. Upregulation of EZH2 in EC tissues correlates with poor prognosis of patients with EC. (A) H&E staining of tumor tissues of EC patients (n = 26) and matched normal tissue control (n = 26). (B) EZH2 mRNA levels of tumor tissues of EC patients (EC, n = 543) and matched normal tissue control (Normal, n **=** 23) from the TCGA database. ****P* < 0.001 vs. matched normal tissue control. (C) Kaplan–Meier plots of overall survival of patients with EC, stratified by EZH2 expression. Data were obtained from the TCGA database; log-rank test, *P* = 0.0005.

Fig. S2. **Knockdown of EZH2 has no effect on apoptosis of EC cells.** (A) Relative mRNA expression levels of EZH2 normalized to GAPDH analyzed by quantitative real-time PCR in scrambled control (Scr) or EZH2 knockdown ISK and KLE cells. Data shown are mean ± SD (n = 3). ****P* < 0.001, ***P* < 0.01 vs. Scr control. (B) Flow cytometric apoptosis assay of ISK and KLE cells with or without EZH2 knockdown.

Fig. S3. **TCF3 regulates p21 expression and its expression levels correlate with EC** prognosis. (A) Diagram showing positions of ChIP primers at the p21 promoter (*top*). ChIP-qPCR analysis of H3K27me3 and H3K27me2 enrichment on p21 promoter (*bottom*). (B) Immunoblots of EZH2 and p53 protein in Scr or EZH2 knockdown ISK and KLE cells. GAPDH served as a loading control. (**C**) Effect of enforced expression of EZH2 on TCF3 protein levels in ISK (Left) and KLE (KLE) cells. GAPDH served as a loading control. (D) Immunoblots of EZH2 and H3K27me3 protein in Scr or EZH2 knockdown ISK cells. GAPDH and histone H3 served as loading controls. (E) Kaplan–Meier plots of overall survival of EC patients stratified by TCF3 expression. Data were obtained from TCGA database; log-rank test, *P* = 0.0001.

Fig. S4. The EZH2/TCF3/p21 axis promotes EC progression. (**A**) Relative mRNA expression levels of EZH2, TCF3 and p21 in xenograft tumors excised from mice analyzed by quantitative real-time PCR in scrambled (Scr) control, EZH2-KD, TCF3-KD, and EZH2-KD + TCF3-KD groups. GAPDH was used as an endogenous control. Data shown are mean ± SD (n = 3). ***P* < 0.01 vs. indicated control. (**B**) The body weights of mice carrying xenograft tumors measured every two days after post-implantation in scrambled (Scr) control, EZH2-KD, TCF3-KD, and EZH2-KD + TCF3-KD mice. (**C**) H&E and immunohistochemical (IHC) staining of EZH2, TCF3, p21, H3K27me3, and Ki67 protein in sections from xenograft tumor tissues excised from scrambled (Scr) control, EZH2-KD, TCF3-KD, and EZH2-KD + TCF3-KD groups (*left*). Total IHC score of stained proteins from scrambled (Scr) control, EZH2-KD, TCF3-KD, and EZH2-KD + TCF3-KD groups. ***P* < 0.001 vs. indicated control (*right*). Scale bars, 50 μm.

Fig. S5. Identification of DNA methyltransferases at the TCF3 promoter. (**A**) CpG islands (Island 1, 2, and 3) at the human TCF3 promoter region (~2.5 kb upstream of the transcriptional start site, TSS) were predicated using online MethPrimer software. The numbers at the bottom represent the positions of the CpG dinucleotides relative to the TSS. (**B**) Immunoblots of H3K27me3 protein in ISK and KLE cells after 5 μM 5-Aza treatment for 7 days. Histone H3 served as a loading control. (**C**) Enrichment of H3K27me3 on TCF3 promoter detected by ChIP-qPCR in ISK cells after treatment with vehicle or 5 μM 5-Aza for 7 days. Data shown are mean ± SD (n = 3). ***P* <0.01 vs. IgG control. The same primers were used for ChIP as in Fig. 3C. (D) The effect of 5-Aza on cellular proliferation measured by CCK8 assay. ISK cells were treated with vehicle or 5 μM 5-Aza for 4 days. Data shown are mean ± SD (n = 3). ****P* <0.001 vs. vehicle control. (**E**) The DNA methylation of three predicated CpG islands at the TCF3 promoter analyzed by bisulfite sequencing PCR (BSP). Each row shows the methylation status of individual CpG dinucleotides determined by sequence analysis of 15 representative individual cloned polymerase chain reaction (PCR) products of the TCF3 genes after bisulfite modification from ISK cells. (**F**) Relative mRNA expression levels of DNMTs, TCF3, and p21 in DNMT1, DNMT3A, and DNMT3B knockdown ISK cells. GAPDH was used as an endogenous control. Data shown are mean ± SD (n = 3). ***P* <0.01 vs. Scr control. (G) Immunoblots of DNMT1 or DNMT3A, and TCF3 and p21 in DNMT1 knockdown and in DNMT3A knockdown ISK cells. GAPDH served as a loading control.

Fig. S6. DNMT3B accelerates the proliferation of EC cells by directly repressing TCF3 *in vitro.* (A, B) Cell proliferation measured by CCK8 (A) and colony formation (B) assay after DNMT3B knockdown in ISK and KLE cells. ***P* < 0.01 vs. Scr. (**C**) Immunoblots of DNMT3B, TCF3, p21, and EZH2 proteins following knockdown of DNMT3B and TCF3 individually or simultaneously in ISK (*left*) and KLE (*right*) cells. GAPDH was used as an internal loading control. (D) Relative mRNA expression levels of EZH2, DNMT3B, TCF3, or p21 in ISK and KLE cells from the scrambled (Scr) control, DNMT3B-KD, TCF3-KD, and Double-KD (DNMT3B-KD + TCF3-KD) groups. GAPDH was used as an endogenous control. Data shown are mean ± SD (n = 3*).* ***P* < 0.01 vs. Scr control. (**E, F**) Cellular proliferation measured by CCK8 (**E**) and colony formation assay (**F**) following knockdown of DNMT3B and TCF3 individually or simultaneously in ISK and KLE cells. ******P* < 0.001, ***P* < 0.01 vs. Scr. (**G**) IHC staining of DNMT3B protein in matched human normal and EC tissues (*left*). Representative micrographs are shown in original magnification (400×). Scale bars, 50 μm. Total IHC score of DNMT3B in matched human normal and EC tissues (n = 26). ****P* < 0.001 vs. Normal control (*right*). (H) DNMT3B mRNA levels in tumor tissues of EC patients (EC, n = 543) and matched normal tissue controls (Normal, n **=** 23) from the TCGA database. ****P* < 0.001 vs. matched normal tissue control. (I) Kaplan–Meier plots of overall survival of EC patients, stratified by DNMT3B expression. Data were obtained from the TCGA database; log-rank test, *P* = 0.0057. (**J**) Pearson correlation scatter plot of H scores of DNMT3B and TCF3 (*left*) or of DNMT3B and p21 (*right*) in human EC (n = 26).

Fig. S7. Synergistic effect of the combination of GSK126 and 5-Aza in EC. (A) Body weights of CDX tumor-carrying mice treated with vehicle, GSK126 (100 mg/kg), 5-Aza (2.5 mg/kg), or GSK126 + 5-Aza (Combo) measured every two days post-implantation. (**B**) Western blot analysis of indicated proteins in xenograft tumors excised from mice treated with vehicle, GSK126 (100 mg/kg), 5-Aza (2.5 mg/kg), or GSK126 + 5-Aza (Combo). GAPDH and histone H3 were used as endogenous loading controls. (**C**) H&E staining and immunohistochemical (IHC) staining of EZH2, TCF3, p21, H3K27me3, and Ki67 protein in xenograft tumor tissues excised from mice treated with vehicle, GSK126 (100 mg/kg), 5-Aza (2.5 mg/kg), or GSK126 + 5-Aza (Combo). Scale bars, 20 μm. (**D**) Body weights of PDX tumor-carrying mice treated with vehicle, GSK126 (100 mg/kg), 5-Aza (2.5 mg/kg), or GSK126 + 5-Aza (Combo) measured every two days post-implantation. (**E**) Western blot analysis of indicated proteins in PDX tumors excised from mice treated with vehicle, GSK126 (100 mg/kg), 5-Aza (2.5 mg/kg), or GSK126 + 5-Aza (Combo). GAPDH and histone H3 were used as endogenous loading controls. (**F**) H&E staining and immunohistochemical (IHC) staining of EZH2, TCF3, p21, H3K27me3, and Ki67 protein in PDX tumor tissues excised from mice treated with vehicle, GSK126 (100 mg/kg), 5-Aza (2.5 mg/kg), or GSK126 + 5-Aza (Combo). Scale bars, 20 μm.

Fig. S8. Ectopic overexpression of TCF3 in EC cells reverses the effect of EZH2/DNMT3B on cell proliferation. **(A**) Immunoblots of EZH2, TCF3 and p21 protein in empty vector (Vector), EZH2-OE (EZH2 overexpression), TCF3-OE (TCF3 overexpression), or Double-OE (EZH2-OE + TCF3-OE) ISK cells. GAPDH served as a loading control. (**B)** Relative mRNA expression levels of EZH2, TCF3 and p21 in ISK cells were analyzed by quantitative real-time PCR in empty vector (Vector), EZH2-OE (EZH2 overexpression), TCF3-OE (TCF3 overexpression), or Double-OE (EZH2-OE + TCF3-OE) groups. GAPDH was used as an endogenous control. Data shown are mean ± SD (n = 3). ***P* < 0.01 vs. indicated control. (**C, D**) Cellular proliferation measured by CCK8 (**C**) and colony formation (**D**) assays following overexpression of EZH2 or TCF3 individually or simultaneously in ISK cells. ***P* < 0.01 vs. indicated control. **(E**) Immunoblots of DNMT3B, TCF3 and p21 protein in empty vector (Vector), DNMT3B-OE (DNMT3B overexpression), TCF3-OE (TCF3 overexpression), or Double-OE (DNMT3B-OE + TCF3-OE) ISK cells. GAPDH served as a loading control. (**F**) Relative mRNA expression levels of DNMT3B, TCF3 and p21 in ISK cells were analyzed by quantitative real-time PCR in empty vector (Vector), DNMT3B-OE (DNMT3B overexpression), TCF3-OE (TCF3 overexpression), or Double-OE (DNMT3B-OE + TCF3-OE) groups. GAPDH was used as an endogenous control. Data shown are mean ± SD (n = 3). ***P* < 0.01 vs. indicated control. (**G, H**) Cellular proliferation measured by CCK8 (**G**) and colony formation (**H**) assays following overexpression of DNMT3B or TCF3 individually or simultaneously in ISK cells. ***P* < 0.01 vs. indicated control.

Fig. S9. Synergistic effect of combined treatment with EPZ-6438 and 5-Aza in EC cells. (A) Drug dose-response matrix for growth inhibition of ISK (*left*) and KLE (*right*) cells following 7-day culture with EPZ-6438 plus 5-Aza. Color gradation indicates percentage viability at the indicated dose combination. Combination index (CI) plots for 5 μM 5-Aza (*top*) or EPZ-6438 (*bottom*) with graded doses of 5-Aza or GSK126 in ISK and KLE cells. (B) Immunoblots of TCF3, p21, and EZH2 protein in ISK (*left*) and KLE (*right*) cells following treatment with EPZ-6438 (5 μM) or 5-Aza (5 μM), or combination for 7 days. GAPDH was used as a loading control. (C, D) CCK8 (C) and colony formation (D) assay were performed to evaluate the effect of the combination treatment with EPZ-6438 and 5-Aza on cell proliferation of ISK and KLE cells. ***P* < 0.01 vs. indicated control.

Fig. S10. Synergistic effect of combined treatment with EPZ-6438 and 5-Aza in vivo.

(A, B, C, D) Photograph (**A**), tumor volumes (**B**), tumor masses **(C)** and body weight (**D**) of ISK cell-derived xenograft tumors excised from mice treated with vehicle, EPZ-6438 (100 mg/kg), 5-Aza (2.5 mg/kg) or combination. Data are presented as the means ± SD; ***P* < 0.01, ****P* < 0.001 vs. indicated control. (**E**) Western blot analysis of indicated proteins in CDX tumors excised from mice treated with vehicle, EPZ-6438 (100 mg/kg), 5-Aza (2.5 mg/kg), or Combo (EPZ-6438 + 5-Aza). GAPDH and histone H3 were used as endogenous loading controls. (**F, G, H, I**) Photograph (**F**), tumor volumes (**G**), tumor masses (**H**), and bodyweight **(I)** of patient-derived xenograft tumors excised from mice treated with EPZ-6438 (100 mg/kg) or 5-Aza (2.5 mg/kg) or combination. Data are presented as the means ± SD; **P* <0.05, ***P* <0.01, ****P* <0.001 vs. indicated control. (**J**) Western blot analysis of indicated proteins in PDX tumors excised from mice treated with vehicle, EPZ-6438 (100 mg/kg), 5-Aza (2.5 mg/kg), or Combo (EPZ-6438 + 5-Aza). GAPDH and histone H3 were used as endogenous loading controls.
