# Supplementary material for: TCF3 is epigenetically silenced by EZH2 and DNMT3B and functions as a tumor suppressor in endometrial cancer
Source: Cell Death Differ. 2021 Jun 26;28(12):3316–28. doi: 10.1038/s41418-021-00824-w (PMC8630057; doi:10.1038/s41418-021-00824-w)
Supplement: Supplementary file 2 — Supplemental figures and tables [file 41418_2021_824_MOESM2_ESM.doc]

**Supplementary figures and tables**


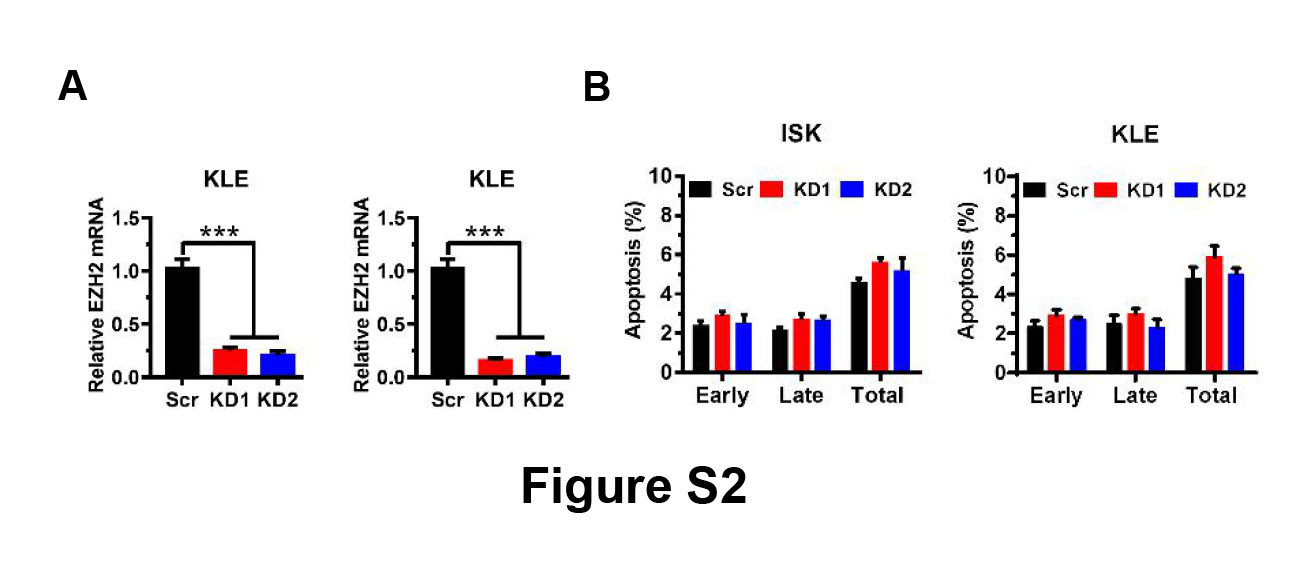


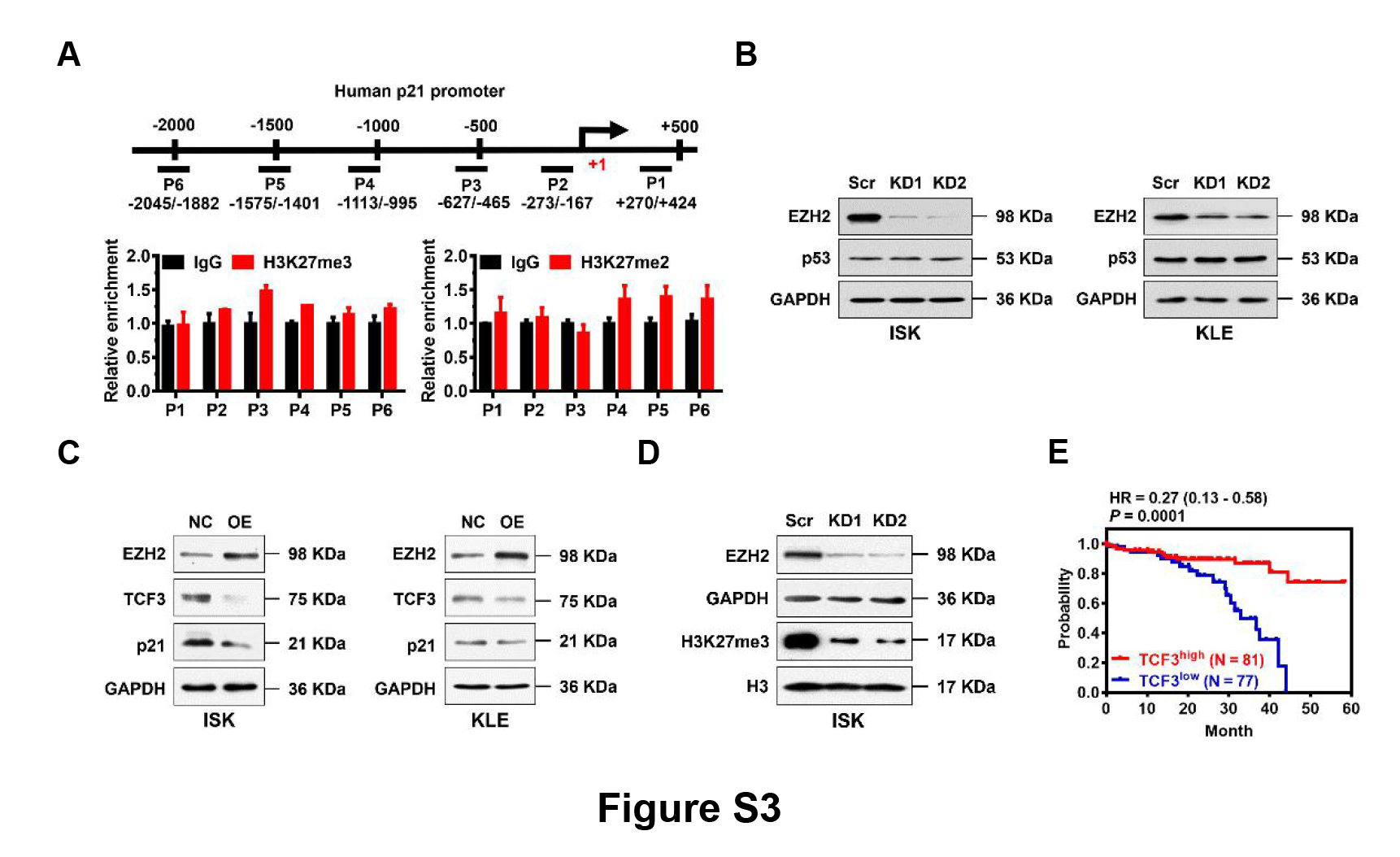


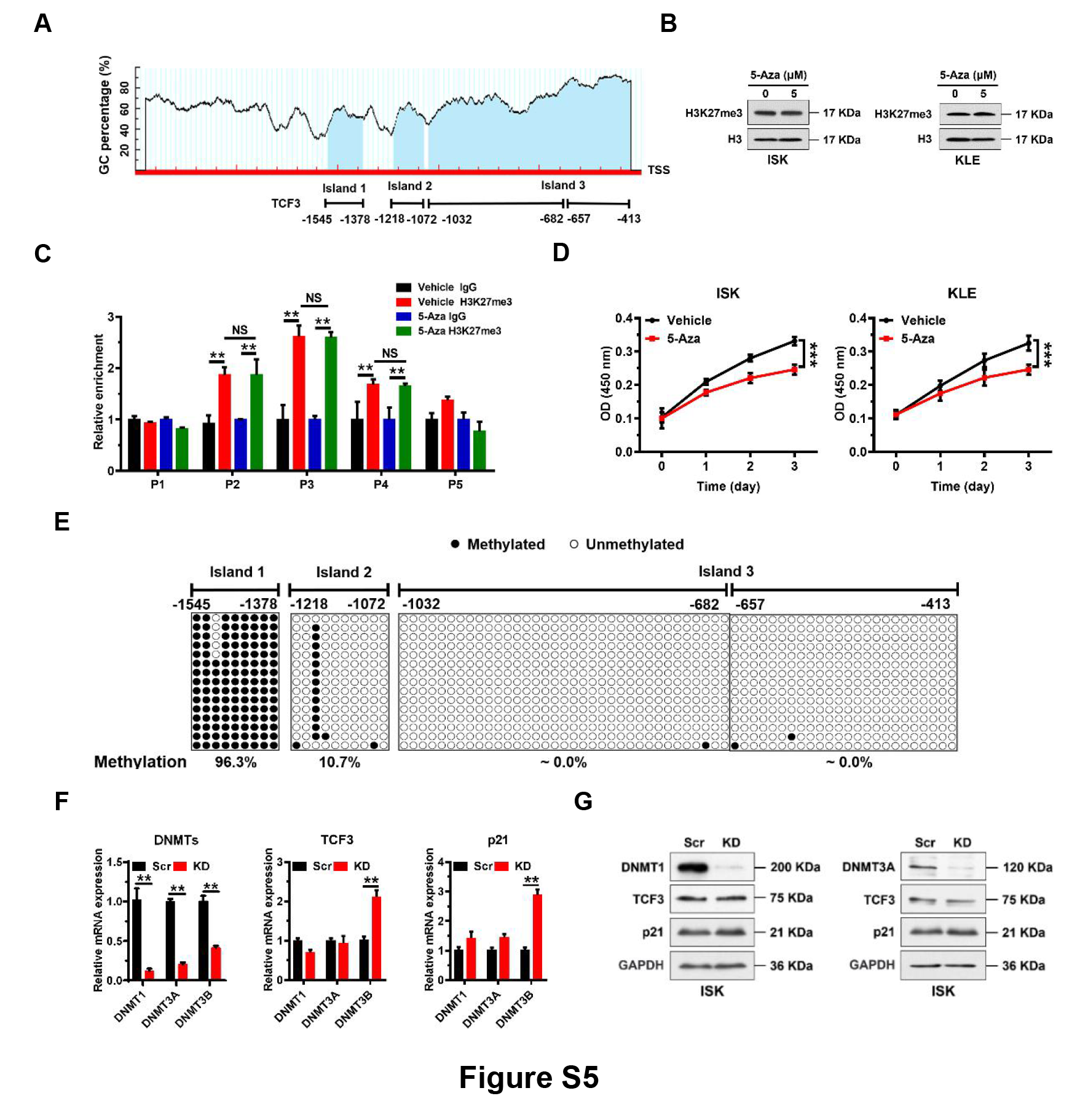


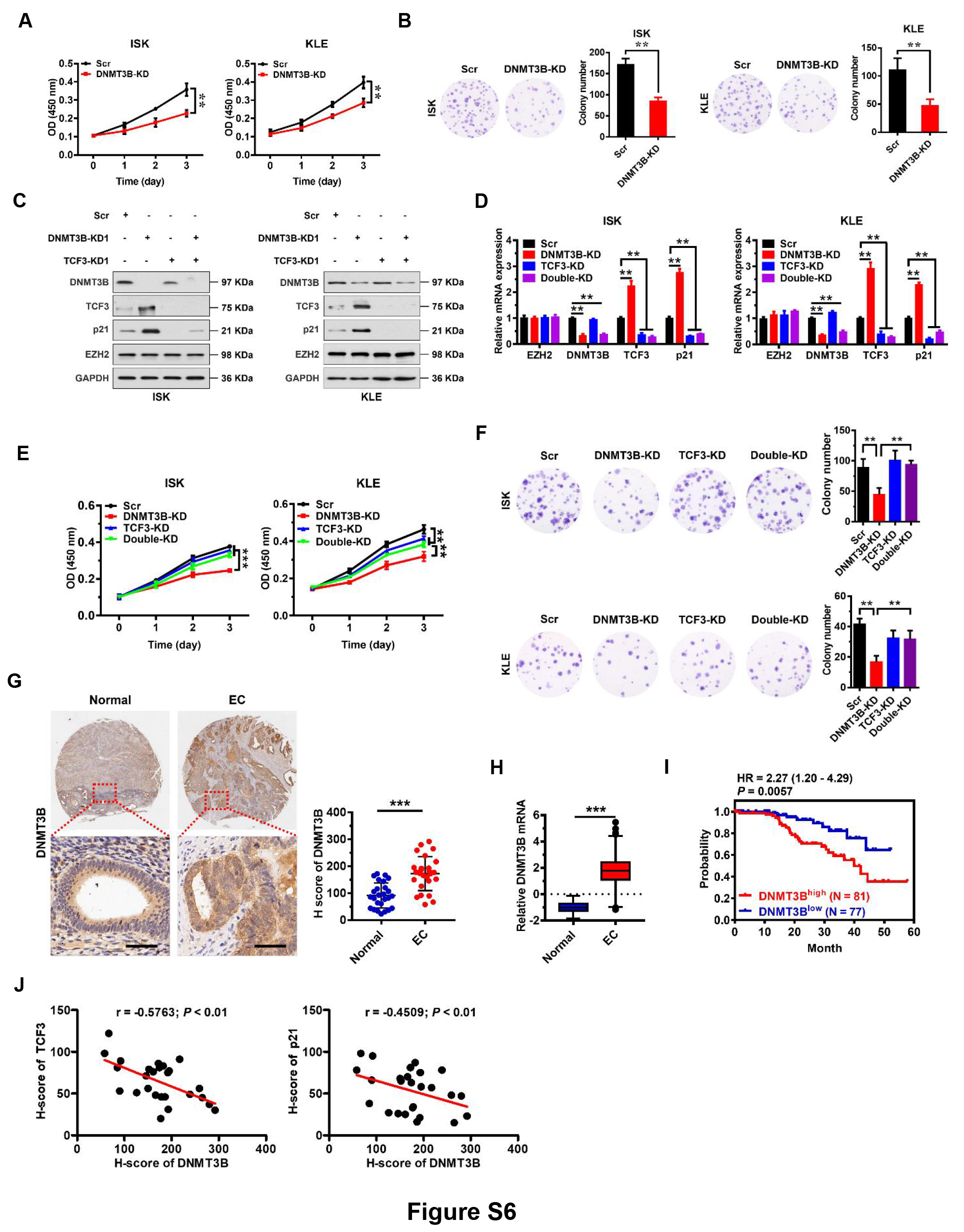


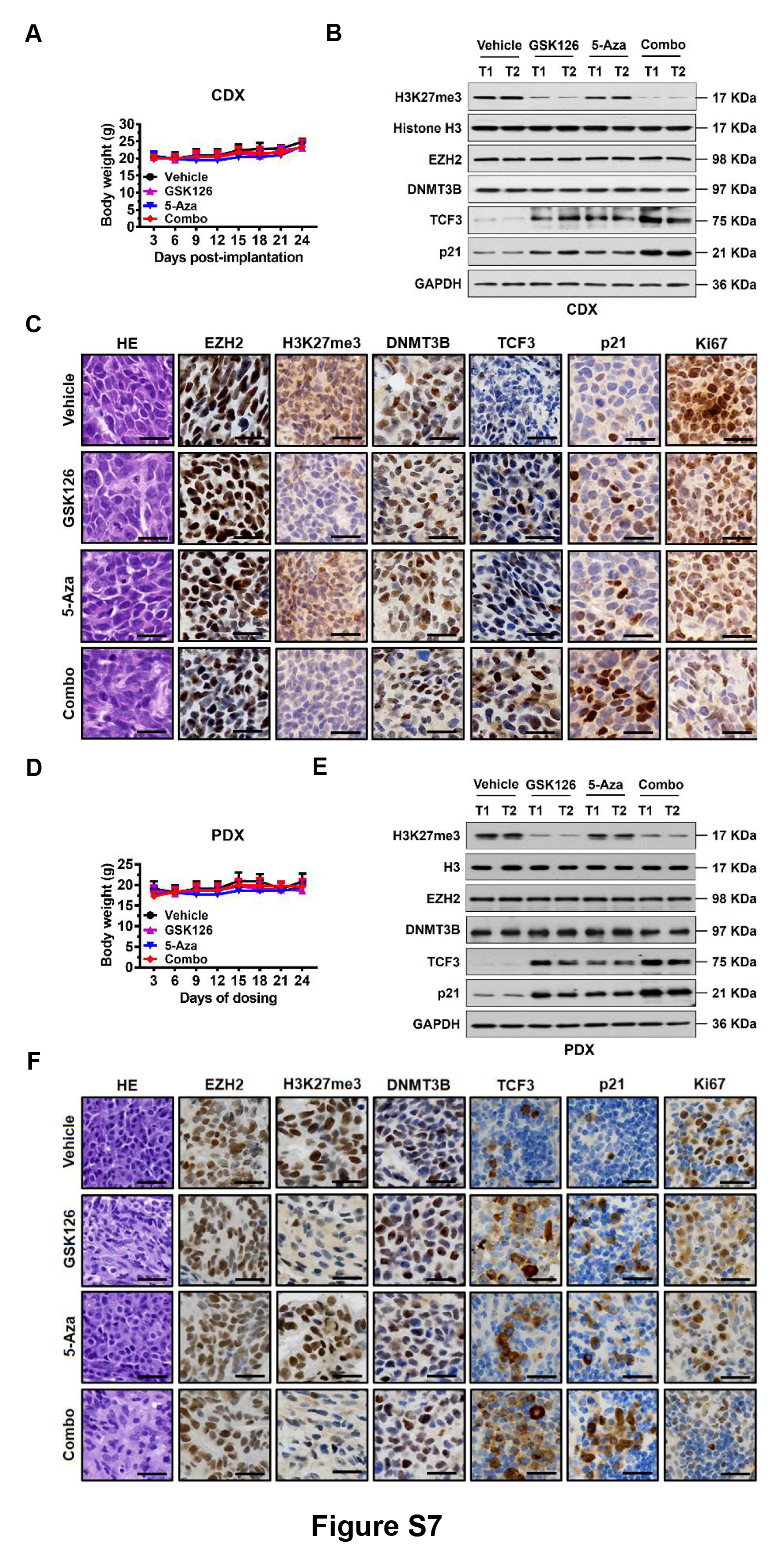


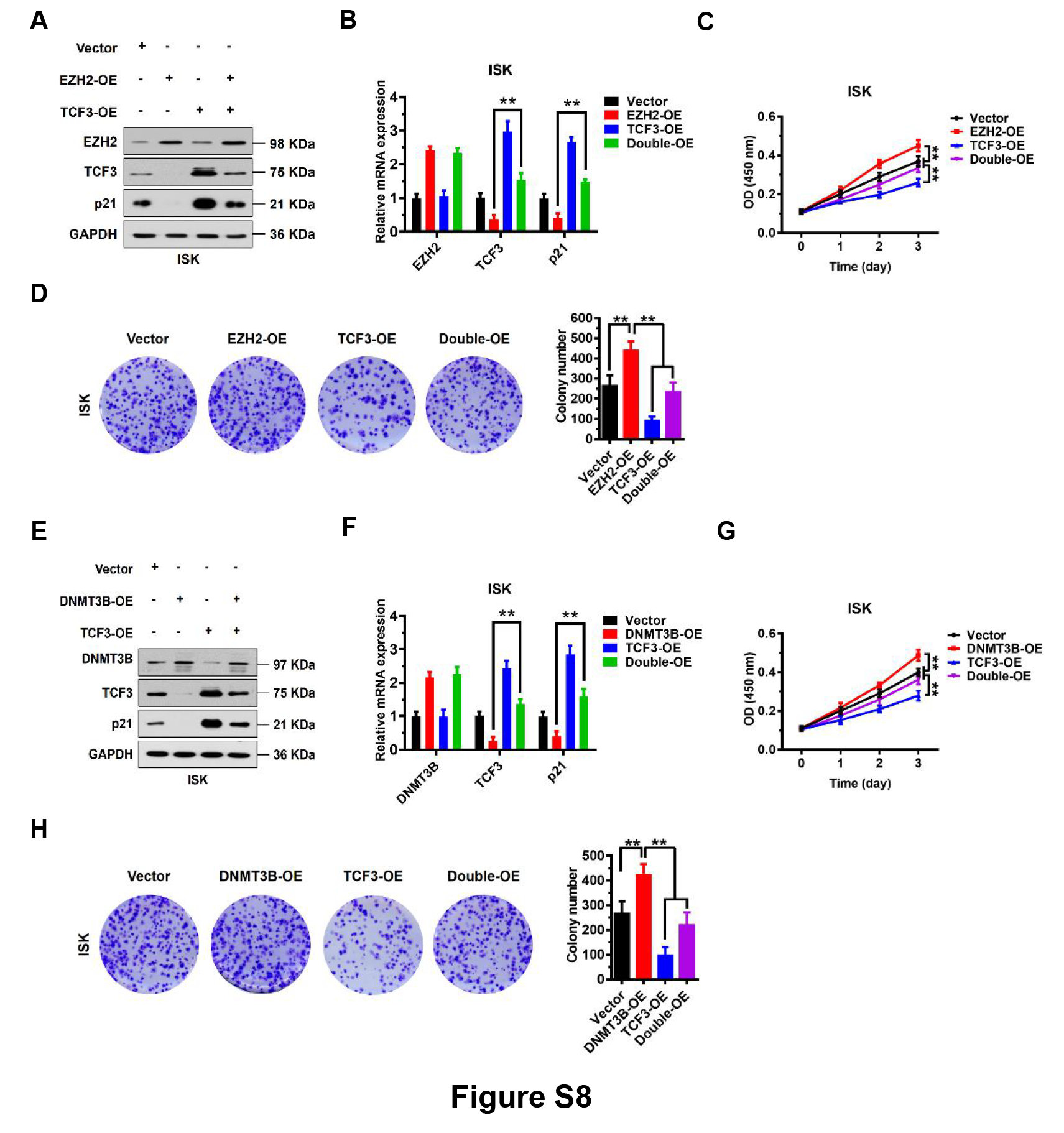


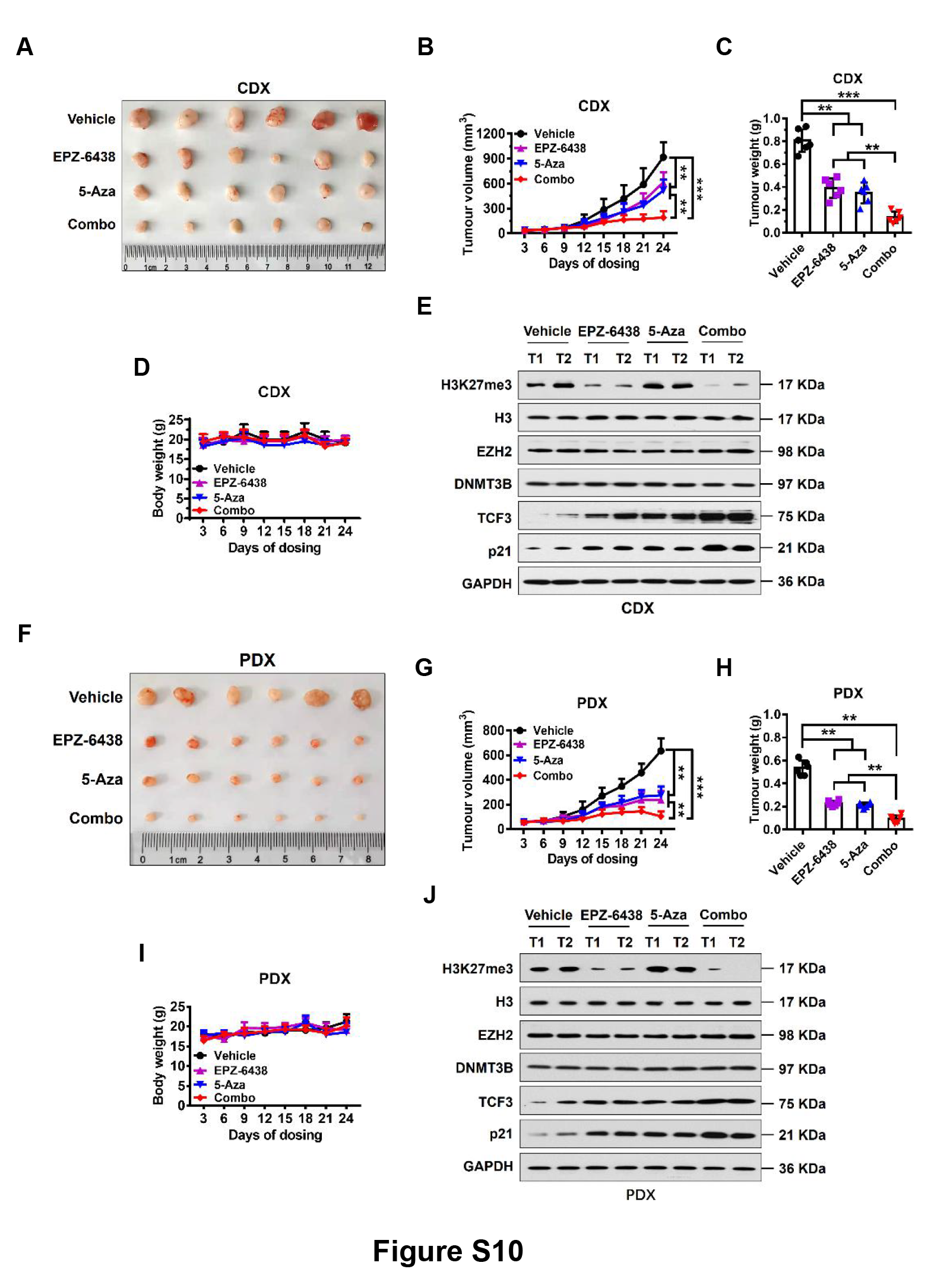


**Table S1. Clinicopathologic characteristics and EZH2 / DNMT3B expression in EC patients**

| **Variable** | **cases** | **H-score of EZH2** | | **H-score of DNMT3B** | |
| --- | --- | --- | --- | --- | --- |
|  |  | Mean ± SEM | *P* value^a^ | Mean ± SEM | *P* value^a^ |
| Age, years |  |  |  |  | 0.74 |
| ≤ 55 | 14 | 155.1 ± 17.0 | 0.15 | 177.0 ± 19.4 |  |
| > 55 | 12 | 108.0 ± 27.8 |  | 168.0 ± 15.1 |  |
| Myometrial invasion^b^ |  |  |  |  |  |
| < 1/2 | 20 | 109.8 ± 14.5 | 0.05 | 154.9 ± 12.1 | 0.005 |
| ≥ 1/2 | 6 | 212.2 ± 37.3 |  | 232.8 ± 22.7 |  |
| Lymph node status^b^ |  |  |  |  | 0.11 |
| N_0_ | 23 | 129.4 ± 16.9 | 0.51 | 180.1 ± 12.8 |  |
| N_1-3_ | 3 | 163.7 ± 60.2 |  | 117.3 ± 29.9 |  |
| Distant metastasis^b^ |  |  |  |  |  |
| No | 25 | 127.6 ± 15.7 | NA | 172.7 ± 12.9 | NA |
| Yes | 1 | 277.0 ± 0 |  | 177.0 ± 0 |  |
| FIGO stage^b^ |  |  |  |  | 0.02 |
| Ⅰ-Ⅱ | 18 | 101.7 ± 18.1 | 0.005 | 149.9 ± 15.8 |  |
| Ⅲ-Ⅳ | 8 | 193.2 ± 20.8 |  | 209.6 ± 14.8 |  |

^a^ Student t test was used to compare the two parameters in each category.

^b^ Myometrial invasion, lymph node status, distant metastasis, and FIGO stage were classified according to the 7th edition of the American Joint Committee on Cancer staging system for endometrial cancer.

**Table S2. Predicted Transcription factors for CDKN1A (p21^WAF1/Cip1^) promoter determined by PROMO (TRANSFAC Version 8.3)^c^**

| **Transcription factors** | **Binding motif** | **Position** |
| --- | --- | --- |
| TP53 | GGGCATG | -1317 ~ -1311; -2779 ~ -2773 |
| CEBPA | GAGCAA | -2861 ~ -2856 |
| Sp1 | GGGCGG | -48 ~ -43; -38 ~ -33 |
| IRF1 | GTGGGGAAA | -208 ~ -200 |
| c-Myc | CAGCTG | -92 ~ -87; -349 ~ -344; -1341 ~ -1336 |
| E2F1 | TCCCGCC | -12 ~ -6 |
| ELK1 | GGAAG | -157 ~ -153; -622 ~ -618; -1328 ~ -1324 |
| TCF3 | GCCAGAT | -229 ~ -223 |
| E47 | GCCAGAT | -229 ~ -223 |
| E12 | GCCAGAT | -229 ~ -223 |
| VDR | GGGT | -15 ~ -12; -336 ~ -339; -509 ~ -506 |
| MZF1 | GTGGGGA | -208 ~ -202; -819 ~ -813 |
| p300 | AGTCC | -920 ~ -916; -1137 ~-1133; -1925 ~-1921 |
| NF1 | TTGGC | -991 ~ -987; -2228 ~ -2224 |
| MyoD1 | CCAGGTGC | -494 ~ -487 |

^c^Reference:

1. Xavier Messeguer, Ruth Escudero, Domènec Farré, Oscar Nuñez, Javier Martínez, M.Mar Albà. PROMO: detection of known transcription regulatory elements using species-tailored searches. *Bioinformatics* **18**, 333-334 (2002).
2. Domènec Farré, Romà Roset, Mario Huerta, José E. Adsuara, Llorenç Roselló, M.Mar Albà, Xavier Messeguer. Identification of patterns in biological sequences at the ALGGEN server: PROMO and MALGEN. *Nucleic. Acids Res*. **31**, 3651-3653 (2003).

**Table S3. qPCR primers**

| **Gene symbol** | **Direction** | **Sequence (5’-3’)** |
| --- | --- | --- |
| EZH2 | Forward | AATCAGAGTACATGCGACTGAGA |
|  | Reverse | GCTGTATCCTTCGCTGTTTCC |
| CDKN2A | Forward | CTTCCTGGACACGCTGGTG |
|  | Reverse | AATCGGGGATGTCTGAGGGA |
| CDKN2B | Forward | ACGGAGTCAACCGTTTCGGGAG |
|  | Reverse | GGTCGGGTGAGAGTGGCAGG |
| CDKN2C | Forward | ACTGGTTTCGCTGTCATTCA |
|  | Reverse | GCAGGTTCCCTTCATTATCC |
| CDKN2D | Forward | GGACCCTCAGCCTTGTTTGT |
|  | Reverse | GGTGGTTTAGCAGAGAC |
| CDKN1A | Forward | TACCCTTGTGCCTCGCTCAG |
|  | Reverse | CGGCGTTTGGAGTGGTAGA |
| CDKN1B | Forward | GGAGCAATGCGCAGGAATAA |
|  | Reverse | TGGGGAACCGTCTGAAACAT |
| CDKN1C | Forward | ATCCACGATGGAGCGTCTTG |
|  | Reverse | TCGTAATCCCAGCGGTTCTG |
| TP53 | Forward | GAGGTTGGCTCTGACTGTACC |
|  | Reverse | TCCGTCCCAGTAGATTACCAC |
| TAp63 | Forward | CAGGACTCGGACCTGAGTGA |
|  | Reverse | GAGGAGCCGTTCTGAATCTG |
| DeltaNp63 | Forward | CTGGAAAACAATGCCCAGAC |
|  | Reverse | GAGGAGCCGTTCTGAATCTG |
| TP73 | Forward | GACGAGGACACGTACTACCTT |
|  | Reverse | CTGCCGATAGGAGTCCACCA |
| RB1 | Forward | ATCACAGCGATACAAACTTGGAG |
|  | Reverse | AGCGCACGCCAATAAAGACA |
| E2F1 | Forward | AGCTGGACCACCTGATGAAT |
|  | Reverse | GAGGGGCTTTGATCACCATA |
| E2F2 | Forward | AGACTCGGTATGACACTTC |
|  | Reverse | CACTGGATGTTGTTCTTGG |
| PTEN | Forward | TGAGTTCCCTCAGCCGTTACCT |
|  | Reverse | GAGGTTTCCTCTGGTCCTGGTA |
| CDK1 | Forward | CTGGGGTCAGCTCGTTACTC |
|  | Reverse | TCCACTTCTGGCCACACTTC |
| CDK6 | Forward | GGATAAAGTTCCAGAGCCTGGAG |
|  | Reverse | GCGATGCACTACTCGGTGTGAA |
| CDK7 | Forward | GCACACCAACTGAGGAACAGTG |
|  | Reverse | AAGTCGTCTCCTGCTGCACTGA |
| CCNB1 | Forward | GACCTGTGTCAGGCTTTCTCTG |
|  | Reverse | GGTATTTTGGTCTGACTGCTTGC |

-Continued-

| **Gene symbol** | **Direction** | **Sequence (5’-3’)** |
| --- | --- | --- |
| CCNB2 | Forward | CAACCAGAGCAGCACAAGTAGC |
|  | Reverse | GGAGCCAACTTTTCCATCTGTAC |
| CCNC1 | Forward | GCAGAAAGATGCCAGGCAATGG |
|  | Reverse | CTCTCATCGAAATTCTTCCACTGC |
| CCND1 | Forward | TCTACACCGACAACTCCATCCG |
|  | Reverse | TCTGGCATTTTGGAGAGGAAGTG |
| CCNE1 | Forward | TGTGTCCTGGATGTTGACTGCC |
|  | Reverse | CTCTATGTCGCACCACTGATACC |
| CCNF1 | Forward | CTGCGTCTTGAGCCTCCATAAG |
|  | Reverse | CCTGGCTGATTTCTCCATAGCG |
| HOXA10 | Forward | ACAAGAAATGTCAGCCAGAAAGG |
|  | Reverse | GATGAGCGAGTCGACCAAAAA |
| KRAS | Forward | AGTGCCTTGACGATACAGC |
|  | Reverse | ACAAAGAAAGCCCTCCC |
| PCNA | Forward | ACACTAAGGGCCGAAGATAACG |
|  | Reverse | ACAGCATCTCCAATATGGCTGA |
| CHEK2 | Forward | TCTCGGGAGTCGGATGTTGAG |
|  | Reverse | CCTGAGTGGACACTGTCTCTAA |
| CCNG1 | Forward | GCCACTTGAAAGGAGAAATAGC |
|  | Reverse | AATGATAGACAATGCCAACACAG |
| CDK5RAP1 | Forward | ATGGCTGCCAGATGAATGTGA |
|  | Reverse | CTCTTGGAGGTTACTGGTCCG |
| CEBPA | Forward | GGACCCTCAGCCTTGTTTGT |
|  | Reverse | GGTGGTTTAGCAGAGAC |
| SP1 | Forward | AGTTCCAGACCGTTGATGGG |
|  | Reverse | GTTTGCACCTGGTATGATCTGT |
| IRF-1 | Forward | ATGCCCATCACTCGGATGC |
|  | Reverse | CCCTGCTTTGTATCGGCCTG |
| c-Myc | Forward | CCTGGTGCTCCATGAGGAGAC |
|  | Reverse | CAGACTCTGACCTTTTGCCAGG |
| ELK1 | Forward | CACTTCTGGAGCACCCTGAG |
|  | Reverse | GGCCATCCACGCTGATAGAA |
| TCF3 | Forward | ACTCCTACAGTGGGCTAGGG |
|  | Reverse | TCTTCTCCTCCTCCGAGTGG |
| E12 | Forward | GAGGCCTTTAAGGAGCTGGG |
|  | Reverse | ATGAGCAGTTTGGTCTGGGG |
| E47 | Forward | GAGAAAGACCTGAGGGACCG |
|  | Reverse | GGCCTCGTTAATATCCCGCA |
| VDR | Forward | TGGAGACTTTGACCGGAACG |
|  | Reverse | GGGCAGGTGAATAGTGCCTT |

-Continued-

| **Gene symbol** | **Direction** | **Sequence (5-3’)** |
| --- | --- | --- |
| MZF1 | Forward | GCTGCTGCCCTAGTAGATGG |
|  | Reverse | CCCAGTGGTGATTCCTGCAT |
| p300 | Forward | GCAGTGTGCCAAACCAGATG |
|  | Reverse | GGGTTTGCCGGGGTACAATA |
| NF1 | Forward | AAAACCAGCGGAACCTCCTT |
|  | Reverse | TTCAGTAGGGAGTGGCAAGT |
| MyoD1 | Forward | GCCACAACGGACGACTTCTA |
|  | Reverse | AGTGCTCTTCGGGTTTCAGG |
| EED | Forward | CTGTAGGAAGCAACAGAGTTACC |
|  | Reverse | CATAGGTCCATGCACAAGTGT |
| SUZ12 | Forward | TTGCAGCTTACGTTTACTGGTT |
|  | Reverse | GGAACTTGCCTTATTGGACAACT |
| DNMT1 | Forward | AGGAGGGCTACCTGGCTAAA |
|  | Reverse | GCTTAGCCTCTCCATCGGAC |
| DNMT3A | Forward | CCTGTGGGAGCCTCAATGTT |
|  | Reverse | CCACACACTCCACGCAAAAG |
| DNMT3B | Forward | TGGAATAGGGGACCTCGTGT |
|  | Reverse | ATGCCAGACATAGCCTGTCG |
| GAPDH | Forward | GAGCCACATCGCTCAGACAC |
|  | Reverse | CATGTAGTTGAGGTCAATGAAGG |

**Table S4. shRNAs used in lentivirus expression vector construction for gene knockdown**

| **shRNAs** | **Direction** | **Sequence (5-3’)** |
| --- | --- | --- |
| Scramble  (Scr) | Forward | CCGGCCTAAGGTTAAGTCGCCCTCGCTCGAGCGAGGGCGACTTAACCTTAGGTTTTTG |
|  | Reverse | AATTCAAAAACCTAAGGTTAAGTCGCCCTCGCTCGAGCGAGGGCGACTTAACCTTAGG |
| EZH2 sh1  (KD1) | Forward | CCGGGCTAGGTTAATTGGGACCAAACTCGAGTTTGGTCCCAATTAACCTAGCTTTTTG |
|  | Reverse | AATTCAAAAAGCTAGGTTAATTGGGACCAAACTCGAGTTTGGTCCCAATTAACCTAGC |
| EZH2 sh2  (KD2) | Forward | CCGGCCAACACAAGTCATCCCATTACTCGAGTAATGGGATGACTTGTGTTGGTTTTTG |
|  | Reverse | AATTCAAAAACCAACACAAGTCATCCCATTACTCGAGTAATGGGATGACTTGTGTTGG |
| EZH2 sh3  (KD3) | Forward | CCGGCCCAACATAGATGGACCAAATCTCGAGATTTGGTCCATCTATGTTGGGTTTTTG |
|  | Reverse | AATTCAAAAACCCAACATAGATGGACCAAATCTCGAG ATTTGGTCCATCTATGTTGGG |
| EED sh1  (KD4) | Forward | CCGGTCTTGCTAGTAAGGGCACATACTCGAGTATGTGCCCTTACTAGCAAGATTTTTG |
|  | Reverse | AATTCAAAAATCTTGCTAGTAAGGGCACATACTCGAGTATGTGCCCTTACTAGCAAGA |
| EED sh2  (KD5) | Forward | CCGGCGGCTATTCGACAAACCAGTTCTCGAGAACTGGTTTGTCGAATAGCCGTTTTTG |
|  | Reverse | AATTCAAAAACGGCTATTCGACAAACCAGTTCTCGAGAACTGGTTTGTCGAATAGCCG |
| SUZ12 sh1  (KD6) | Forward | CCGGCGGAATCTCATAGCACCAATACTCGAGTATTGGTGCTATGAGATTCCGTTTTTG |
|  | Reverse | AATTCAAAAACGGAATCTCATAGCACCAATACTCGAGTATTGGTGCTATGAGATTCCG |
| SUZ12 sh2  (KD7) | Forward | CCGGGCTGACAATCAAATGAATCATCTCGAGATGATTCATTTGATTGTCAGCTTTTTG |
|  | Reverse | AATTCAAAAAGCTGACAATCAAATGAATCATCTCGAGATGATTCATTTGATTGTCAGC |
| TCF3 sh1  (TCF3-KD1) | Forward | CCGGCCCGGATCACTCAAGCAATAACTCGAGTTATTGCTTGAGTGATCCGGGTTTTTG |
|  | Reverse | AATTCAAAAACCCGGATCACTCAAGCAATAACTCGAGTTATTGCTTGAGTGATCCGGG |
| TCF3 sh2  (TCF3-KD2) | Forward | CCGGCAGCCTCTCTTCATCCACATTCTCGAGAATGTGGATGAAGAGAGGCTGTTTTTG |
|  | Reverse | AATTCAAAAACAGCCTCTCTTCATCCACATTCTCGAGAATGTGGATGAAGAGAGGCTG |

**-Continued**-

| **shRNAs** | **Direction** | **Sequence (5-3’)** |
| --- | --- | --- |
| DNMT1 sh1  (DNMT1 KD) | Forward | CCGGGCCCAATGAGACTGACATCAACTCGAGTTGATGTCAGTCTCATTGGGCTTTTTG |
|  | Reverse | AATTCAAAAAGCCCAATGAGACTGACATCAACTCGAGTTGATGTCAGTCTCATTGGGC |
| DNMT3A sh1  (DNMT3A KD) | Forward | AATTCAAAAACCACCAGAAGAAGAGAAGAATCTCGAGATTCTTCTCTTCTTCTGGTGG |
|  | Reverse | CCGGACACGCAACCAGTGGTTAATACTCGAGTATTAACCACTGGTTGCGTGTTTTTTG |
| DNMT3B sh1  (DNMT3B KD) | Forward | CCGGGACGATGGCTATCAGTCTTACCTCGAGGTAAGACTGATAGCCATCGTCTTTTTTG |
|  | Reverse | AATTCAAAAAGACGATGGCTATCAGTCTTACCTCGAGGTAAGACTGATAGCCATCGTC |

**Table S5. PCR primers used for** ChIP analysis

| **Fragment (s)** | **Direction** | **Sequence (5’-3’)** |
| --- | --- | --- |
| p21-P1 | Forward | GTTTCTGCGGCAGGTGAAT |
|  | Reverse | GGGAGCGTGACCAGGGAT |
| p21-P2 | Forward | TGTGTCCTCCTGGAGAGTGC |
|  | Reverse | CAGTCCCTCGCCTGCGTTG |
| p21-P3 | Forward | TTCCCGGAAGCATGTGACAAT |
|  | Reverse | CACTAGGTCACCTCTCCCAGA |
| p21-P4 | Forward | AGGTAGATGGGAGCGGATAGA |
|  | Reverse | ACCCTCATTTGCAGATGGTTT |
| p21-P5 | Forward | CATTTGACAACCAGCCCTTT |
|  | Reverse | TGGGAGGACACAGTAGCAGA |
| p21-P6 | Forward | CAGCAGATCCTTGCGACAG |
|  | Reverse | CCATGCACTTGAATGTGTACC |
| p21-P7 | Forward | TCTGGGGTCTCACTTCTTGG |
|  | Reverse | GCCTGAAGAAGGAGGATGTG |
| TCF3-P1 | Forward | AGTGCGGTTTCGTAGTCCTC |
|  | Reverse | GTCGAGGGAGACCCAACTTC |
| TCF3-P2 | Forward | CCATCAGACTTTCGCGGCTT |
|  | Reverse | TGTGCGCTTAGTCCATGACG |
| TCF3-P3 | Forward | GCTGTGAGCACCTACTGTGT |
|  | Reverse | CCTCATCCCTCTCCACCTCA |
| TCF3-P4 | Forward | GCCTCAAGCGATCTTCCC |
|  | Reverse | CCTGATTCTTAGTAGTCCGTGTCT |
| TCF3-P5 | Forward | CTCTGCCTCCTGGGTTTA |
|  | Reverse | GCACGCCATTGTACTCC |
| TCF3-P6 | Forward | TGTTGGACACTTGGGCAGTT |
|  | Reverse | CATGACCACTGCTCACCCAT |

**Table S6. The PCR primers used for bisulfite sequencing**

| **Island** | **Direction** | **Sequence (5-3’)** |
| --- | --- | --- |
| Island 1 | Forward | TTGTTGTTTAGGTTGGAGTATAATG |
|  | Reverse | AATTCRAAACCTCTCTAACCAACAC |
| Island 2 | Forward | TTTTATTATGTTGTTTAGGTTGTTTT |
|  | Reverse | AATCTAACTTCCTAATTCTTAATAATCC |
| Island 3-1 | Forward | TTTAAAGTTAGGTTTAAATTTATAGATGT |
|  | Reverse | AAAATCCTAACCATTCRTAATAAAAC |
| Island 3-2 | Forward | GTTTTATTAYGAATGGTTAGGATTTT |
|  | Reverse | TAACTCRACCTCTCRAACACCC |
